# Dye extract of calyces of *Hibiscus sabdariffa* has photodynamic antibacterial activity: A prospect for sunlight‐driven fresh produce sanitation

**DOI:** 10.1002/fsn3.1580

**Published:** 2020-04-21

**Authors:** Hussaini Majiya, Anzhela Galstyan

**Affiliations:** ^1^ Department of Microbiology Ibrahim Badamasi Babangida University Lapai Nigeria; ^2^ Center for Soft Nanoscience University of Münster Munster Germany

**Keywords:** antibacterial, fresh produce, *Hibiscus sabdariffa*, photodynamic inactivation, Photodynamic sanitation, reactive oxygen species

## Abstract

Photodynamic sanitation of fresh produce could help reduce spoilage and disease transmissions where conventional methods of sanitation are not available, and sunlight is available for free. In this study, we evaluated the photostability and photodynamic antibacterial activity of the dye extracts of calyces of *Hibiscus sabdariffa*. The dye extracts were very photostable in water but bleached in acetate‐HCl buffer (pH 4.6), phosphate buffer saline (pH 7.2), and tris base‐HCl buffer (pH 8.6). The photostability correlated with the photodynamic antibacterial activity of the dye extracts. Both the methanol and water dye extracts at the concentration of 0.0625 mg/ml caused complete inactivation of *Bacillus subtilis* (reductions of 8.5 log CFU/ml) within 2 min either with the visible light exposure at 10 mW/cm^2^ or in the dark without the light exposure. Reductions of 4.8 log CFU/ml and 2.2 log CFU/ml of *Escherichia coli* were observed when 1 mg/ml of methanol and water dye extracts were used, respectively, in water with the light exposure at 10 mW/cm^2^ for 20 min. Discussions are included about the ease of the dye extractions of the calyces of *H. sabdariffa* even in water without the need of energy for heating and the suitability of the dye extracts for the fresh produce sanitation. Dye extract of calyces of *H. sabdariffa* has photodynamic and nonphotodynamic antibacterial activity which could be exploited for the development of a low‐tech sunlight‐driven fresh produce sanitation system that is cheap, sustainable, and environmentally friendly.

## Graphical abstract

Dye extract of calyces of *Hibiscus sabdariffa* is photostable in water and the photostability correlated with the photodynamic antibacterial activity. The dye extract has both photodynamic and nonphotodynamic antibacterial activity. The extract could be used as an alternative food‐grade fresh produce sanitizer.

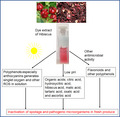

## 1 INTRODUCTION

Food‐grade photosensitizers have been suggested as the most appropriate to be used for the photodynamic sanitation of foods including fresh produce. This is because there would not be the usual health and safety concerns that maybe associated with the use of other types of photosensitizers and chemicals (Ghate, Zhou, & Yuk, 2019; Luksiene & Paskeviciute, 2011; Randazzo, Aznar, & Sánchez, 2016). Conventional methods of fresh produce sanitation are energy, chemical, and operational intensive and, therefore, the new quests for alternative methods such as sunlight‐driven photodynamic sanitation that would be available, cheap, sustainable, environmentally friendly, and applicable in most rural areas where fresh produce are grown especially in developing countries (de Oliveira, Tosati, Tikekar, Monteiro, & Nitin, 2018; Luksiene, Kurilčik, Juršėnas, Radžiutė, & Būda, 2007; Luksiene, Buchovec, & Paskeviciute, 2010; Luksiene, Buchovec, & Viskelis, 2013; Majiya, Chowdhury, Stonehouse, & Millner, 2019).

The lack of postharvest sanitation of fresh produce has both public health and socioeconomic consequences (Aponiene, Paskeviciute, Reklaitis, & Luksiene, 2015; Buchovec et al., 2017; Buchovec, Lukseviciute, Marsalka, Reklaitis, & Luksiene, 2016; Buchovec, Paskeviciute, & Luksiene, 2010; Luksiene, Buchovec, Kairyte, Paskeviciute, & Viskelis, 2012). Many studies have shown links between foodborne outbreaks/diseases and consumption of contaminated fresh produce which are eaten uncooked (de Oliveira et al., 2018; Ghate et al., 2019; Maikai, Elisha, & Baba‐Onoja, 2012; Olaimat & Holley, 2012; Thorn, Pendred, & Reynolds, 2017). However, the main impacts of foodborne diseases are seen in the developing countries because of unsafe water used for cultivating, cleaning, and processing of food, absence of food storage infrastructures, and inadequate/poorly enforced regulatory standards (Chigor et al., 2012; Chigor, Umoh, & Smith, 2010; WHO, 2015). There is little postharvest processing and sanitation of fresh produce in most of the sub‐Saharan Africa and this contributed to about 55% and 72% of fresh produce grown to perish before they can be consumed (Adeoye, Odeleye, Babalola, & Afolayan, 2009; Idah, Ajisegiri, & Yisa, 2007; Olayemi, Adegbola, Bamishaiye, & Daura, 2010). Also, ordinarily, when weather and temperature are considered, the rate of fresh produce spoilage in sub‐Saharan Africa would be 3–4 times than in the Europe. The daily average temperature in sub‐Saharan Africa throughout the year is 30–35°C—this is an optimum temperature range for the growth and proliferation of most microorganisms including fresh produce spoilage and pathogenic bacteria.

Photodynamic effect is the generation of singlet‐oxygen and other reactive oxygen species (ROS) by interaction of a photosensitizer, light of appropriate wavelength and molecular oxygen (Costa et al., 2013; Komagoe, Kato, Inoue, & Katsu, 2011; Lukšiene, 2005; Tavares et al., 2011). Photodynamic sanitation of foods is a process in which the generated singlet‐oxygen and other ROS inactivate pathogens and reduces microbial load of the food to a level considered safe by the public health standards (Aponiene & Luksiene, 2015; Lukšiene, 2005; Luksiene & Brovko, 2013). In developing countries including sub‐Saharan Africa, there are high prospects of successfully establishing cheap, efficient, and sustainable low‐tech photodynamic postharvest sanitation of fresh produce because food‐grade photosensitizers can be sourced locally from the plant materials and wastes, and abundant sunlight can be used to irradiate the photosensitizers (Luksienė & Zukauskas, 2009; Majiya et al., 2019).

*Hibiscus sabdariffa* (also commonly called Roselle) is an annual tropical and subtropical bushy shrub with red or pale green stems and red or pale yellow inflated edible calyces. It is native to west Africa but now grown in other tropical and subtropical countries especially Africa and Asia including Nigeria, Niger, Ghana, Angola, Sudan, Egypt, China, Thailand, Sri Lanka, Malaysia, West India, and also in the Mexico (Abou‐Arab, Abu‐Salem, & Abou‐Arab, 2011; Da‐Costa‐Rocha, Bonnlaender, Sievers, Pischel, & Heinrich, 2014; Ilori & Odukoya, 2005). The red pigmentation of the calyces is due to the high concentration of anthocyanins. Other chemical constituents of the calyces are organic acids, flavonoids, and other polyphenols. The facts that *H. sabdariffa* is not a staple crop, safe for usage as food and medicine, available in abundance, easy extraction of its dye, and easily grown in developing countries (Abou‐Arab et al., 2011; Da‐Costa‐Rocha et al., 2014) make it a potential good candidate as a source of dye extract that can be used as a food‐grade photosensitizer for the development of a low‐tech sunlight‐driven sanitation system for foods including fresh produce in those regions of the world that lacks the conventional systems of fresh produce sanitation.

In this work, we evaluated the dye extracts from the calyces of *H. sabdariffa* for potential usage as a food‐grade photosensitizer. We investigated the photodynamic inactivation of model bacteria using the dye extracts.

## 2 MATERIALS AND METHODS

### 2.1 Calyces of *H. sabdariffa*

The dried pinkish red calyces of *H. sabdariffa* were purchased from the Kure Market, Minna, Niger State, Nigeria. The calyces were used as purchased for the extraction of the dye with no prior preparation and processing.

### 2.2 Solvents and other consumables

All the chemicals, solvents, and other consumable used were of analytical grade. All inorganic chemicals and salts were purchased from Sigma‐Aldrich. Methanol and acetic acid were purchased from Acros Organics. LB broth and agar for culturing bacteria were purchased from Fisher Scientific: Janssen Pharmaceuticalaan. The distilled water used in this work was provided by the Thermo Scientific.

### 2.3 Light source and irradiation conditions

The light source used for the photostability and Photodynamic inactivation (PDI) experiments was a Slide Projector—Zett Royal II afs (Remote Control Leica Projection GmbH Type 508, Zett Gerate), which provides a cool bright light. A 420‐nm light filter was inserted into the projector to cutoff the UV light. Fluence rate of irradiations were measured using a Digital Radiometer (Solar meter, model 9.6 Red light, Solar Tech Inc.). Visible light was used throughout the study and the fluence rates (radiant exposure) were 5–10 mW/cm^2^ at 20–22°C under aerobic conditions. The solvents and buffers used for the photophysical characterization of the dye extracts and PDI were distilled water (dH_2_O), methanol, Phosphate Buffer Saline (PBS; pH 7.2, 6.6, 7.6; 10 mM Na_2_HPO4, 1.8 mM KH_2_PO4, 137 mM NaCl, and 2.7 mM KCl), Acetate‐HCl (AH) Buffer (pH 3.6, 4.6, 5.6; 0.1 M CH_3_COOH and 0.1 M CH_3_COOK), and Tris Base‐HCl (TBH) Buffer (pH 8.6; 0.1 M Tris and 0.1 M HCL).

### 2.4 Bacterial strains

The bacteria strains—*E. coli* Nissle 1917 (a model for Gram‐negative bacteria) and *B. subtilis* DB 104 (a model for Gram‐positive bacteria)—were from the laboratory of Prof. Ulrich Dobrindt, University of Munster, Germany.

### 2.5 Extraction of the dye of calyces of *H. sabdariffa*

Methanol, 0.1% (v/v) acetic acid methanol, and distilled water were used as solvents for the extraction of dye of the calyces of *H. sabdariffa*. The methanol dye extractions of the calyces were carried out under the cold (room temperature 20°C) and hot (60°C) conditions. The water dye extractions were carried out under the cold (room temperature 20°C), hot (60°C), and boiling (100°C) conditions. Five grams of the dried calyces was suspended in 30 ml of the solvent in each of the extraction setup in 100‐ml round‐bottom flasks with continuous shaking at 500 rpm for 2 hr in the first extractions. The dye extracts were then decanted into separate Falcon bottles (50 ml) and 30 ml of the same solvents were added to the extraction flasks maintained under previous conditions for 1 hr for the second extractions. The dye extracts of the second extraction were also decanted into the separate Falcon bottles. The dye extracts were then centrifuged at 11, 500 *g* for 5 min. After the centrifugation, the supernatants were decanted into fresh clean round‐bottom flasks (100 ml). The methanol dye extracts were then concentrated and dried using Rotavapor (BUCHI, Switzerland). The distilled water dye extracts were first frozen using liquid nitrogen and then lyophilized (Christ, Germany) to concentrate and dry. The dried samples were weighed, and the yields of the dye extracts were determined. The samples were then separately dispensed into smaller labeled bottles and stored in the dark under refrigeration for subsequent use.

### 2.6 Photophysical–chemical characterization of the dye extracts of the calyces of *H. sabdariffa*

#### 2.6.1 Absorption spectra

The absorption spectra of the dye extracts (1 mg/ml) of the calyces of *H. sabdariffa* were determined in dH_2_O, AH buffer (pH 3.6, 4.6, 5.6 and 6.6), PBS (pH 7.6), and TBH buffer (8.6) using Agilent 8453 UV‐visible spectrophotometer (Agilent Technologies) and the wavelength range used was from 300 to 800 nm.

#### 2.6.2 pH stability in distilled water

The pH of different concentrations of the dye extract (CM) of the calyces of *H. sabdariffa* and the pH stability were determined and followed for 1 month using a digital pH meter (Seven Compact, Mettler Toledo, USA). The pH of the samples was read and recorded at the beginning of every week until the 4th week.

#### 2.6.3 Photostability and bleaching

Photostability and bleaching of the dye extract (CM) of the calyces of *H. sabdariffa* were determined in distilled water, Acetate Buffer (pH 4.6), PBS (pH 7.2), and Tris Base Buffer (pH 8.6) using Agilent 8453 UV‐visible spectrophotometer (Agilent Technologies). The absorptions (300–800 nm) of the samples were read intermittently every 2 min of the irradiation at 5 mW/cm^2^ until 20th minutes.

### 2.7 Mass spectroscopy of the dye extracts of the calyces of *H. sabdariffa*

Mass spectroscopy of all the dye extracts was determined in methanol and water using MicroTof‐ESI (Bruker Daltonics) and Orbitrap LTQ XL‐ESI (Thermo Fisher Scientific) in order to assess the effect of the temperature conditions and solvents used for the extractions on the compositions of the extracts. Additionally, the fractionization and mass spectroscopy of the dye extract (CM) was done using 6110 HPLC‐MS‐ESI (Agilent Technologies). In all cases, both positive and negative ionization modes of the mass spectroscopy were acquired.

### 2.8 Photodynamic inactivation of model bacteria using the dye extracts of the calyces of *H. sabdariffa*

Photodynamic inactivation (PDI) of the model bacteria (*E. coli* Nissle 1917 and *B. subtilis* DB 104) was investigated with different concentrations of the water and methanol dye extracts of the calyces of *H. sabdariffa* in distilled water (dH_2_O), Acetate‐HCl (AH) Buffer (pH 4.6), PBS (pH 7.2), and Tris Base‐HCl (TBH) Buffer (pH 8.6), and at different times of light illumination. The concentrations of the dye extracts used were 0.0625 mg/ml to 1 mg/ml, light intensity was 10 mW/cm^2^, and illumination times were 2–20 min. For each of the PDI light experiments (dye extract with light illumination), two control experiments were simultaneously setup; dark experiments which were carried out in the presence of dye extracts but without illumination, while “light only” experiments were exposed to light but in the absence of the dye extracts. All experiments were repeated three times. After PDI experiments, the solutions were plated for bacterial enumeration to determine the rate and extent of inactivation. Also, live/dead staining (BacLight Bacterial Viability Kit, Invitrogen) was used according to the manufacturer's instructions to determine the viability of the bacteria after the PDI. Fluorescence images were captured using 40× magnification objective with a Nikon Eclipse Ci microscope (Nikon Corporation Tokyo).

## 3 RESULTS

### 3.1 Effects of the temperature and solvents on the yields and pH of the dye extracts of the calyces of *H. sabdariffa*

The yields of dye extractions of the calyces of *H. sabdariffa* by different temperatures and solvents are shown in Table 1. When solvents are considered, more higher yields were observed in water dye extractions of the calyces (Table 1). As for the temperatures of the extractions, hot temperature gave higher yields compared with the cold temperature (Table 1). Although all the dye extracts irrespective of the solvents and temperatures of extractions are very soluble in water, the pH varies slightly in dH_2_O at the same concentration (Table 1). The methanol dye extracts have lower pH when compared with the water dye extracts (Table 1).

**TABLE 1:** Yields and pH determined in dH_2_O of dye extracts of calyces of *Hibiscus sabdariffa*

Temperature and extraction solvent	Dye extract yield (mg) per 5 g of dried calyces	% yield of dye extracts	pH (1 mg/ml)
CM	1,407.2	28.14	2.85
HM	1,554.4	31.09	2.86
CMA	1,281	25.62	2.76
HMA	1,402.5	28.05	2.76
CW	1,775.8	35.52	3.0
HW	1,987	39.74	3.05
BW	1,514	30.28	3.11

Abbreviations: BW, boiling distilled water dye extract; CM, cold methanol dye extract; CMA, cold acidified methanol dye extract; CW, cold distilled water dye extract; HM, hot methanol dye extract; HMA hot acidified methanol dye extract; HW, hot distilled water dye extract.

### 3.2 Photophysical characterization of the dye extracts of the calyces of *H. sabdariffa*

#### 3.2.1 Effect of buffers/solutions on the absorption spectra and colors of the dye extracts

**FIGURE 1:**
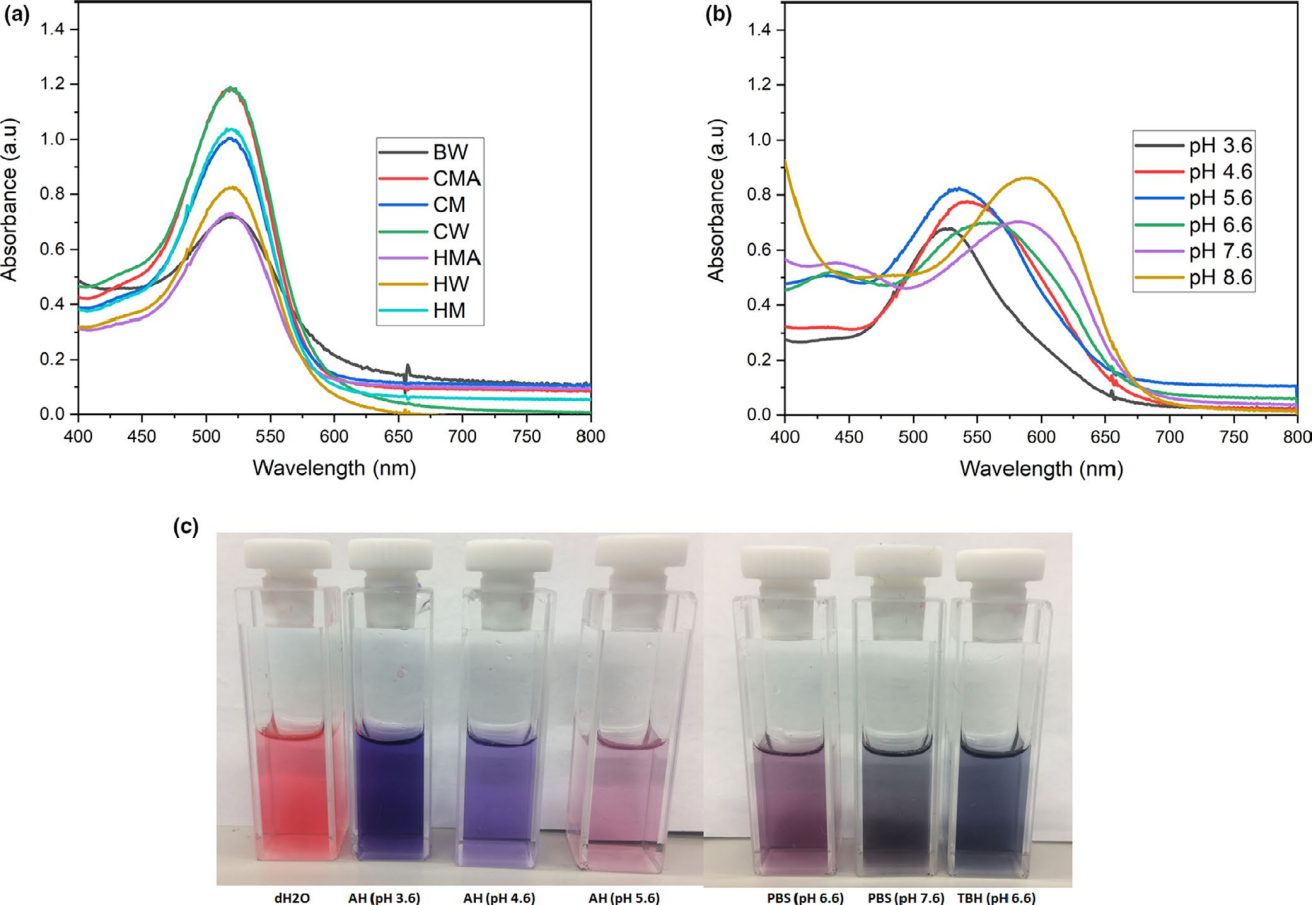
Absorption spectra and colors of dye extracts of calyces of *Hibiscus sabdariffa* in different solutions. (a) Absorption spectra determined in dH_2_O of the dyes extracted by different temperatures and solvents. The absorption peaks for all the samples were at 520 nm. (b) Absorption spectra determined in different buffers and pH of the dye extract (CM). The peaks for pH 3.6, 4.6, 5.6, 6.6, 7.6, and 8.6 were at 530, 538, 536, 559, 583, and 592 nm, respectively. (c) Colors of the dye extract (CM) in dH_2_O and buffers at different pH. AH, acetate‐HCl buffer; BW, boiling distilled water dye extract; CM, cold methanol dye extract; CMA, cold acidified methanol dye extract; CW, cold distilled water dye extract; dH_2_O, distilled water; HM, hot methanol dye extract; HMA, hot acidified methanol dye extract; HW, hot distilled water dye extract; PBS, phosphate buffer saline; TBH, tris base‐HCl buffer

The absorption spectra (Figure 1) of the dye extracts (1 mg/ml) of the calyces of *H. sabdariffa* were determined in distilled water (dH_2_O), Acetate‐HCl (AH) buffer (pH 3.6, 4.6, and 5.6), Phosphate Buffer Saline (PBS; pH 6.6 and 7.6), and Tris base‐HCl (TBH) buffer (8.6). The peak absorption for all the samples in dH_2_O was at 520 nm (Figure 1a). However, the intensity of the absorptions differs, and samples extracted under cold conditions seem to have higher intensity compared with the samples extracted at hot and boiling conditions (Figure 1a). Also, although the absorption peaks for all samples were at 520 nm, the spectrum pattern of the sample extracted under boiling condition (BW) slightly differs with other samples (Figure 1a). The absorption spectra of the dye extract (CM) in different buffers and pH have different absorption peaks and colors (Figure 1b,c). The absorption peaks tend to move toward infrared as the pH increases (Figure 1b). The peaks for pH 3.6, 4.6, 5.6, 6.6, 7.6, and 8.6 were at 530, 538, 536, 559, 583, and 592 nm, respectively (Figure 1b). The changes in colors with different pH are characteristics of the dye anthocyanins. The pH stability of different concentrations of dye extract (CM) in dH_2_O for 1 month is shown in Table 2. The dye extract is very stable in water (Table 2).

**TABLE 2:** The pH stability of the dye extract (CM) in dH_2_O monitored for 1 month

Concentration (mg/ml)	pH (Week 1)	pH (Week 2)	pH (Week 3)	pH (Week 4)
1	2.85	2.82	2.78	2.86
0.5	3.03	3.10	3.06	3.08
0.25	3.26	3.33	3.28	3.35
0.125	3.55	3.55	3.56	3.62
0.0625	3.71	3.72	3.81	3.85

Abbreviation: CM, cold methanol dye extract.

#### 3.2.2 Effects of buffers/solutions on the photostability and bleaching of the dye extracts

**FIGURE 2:**
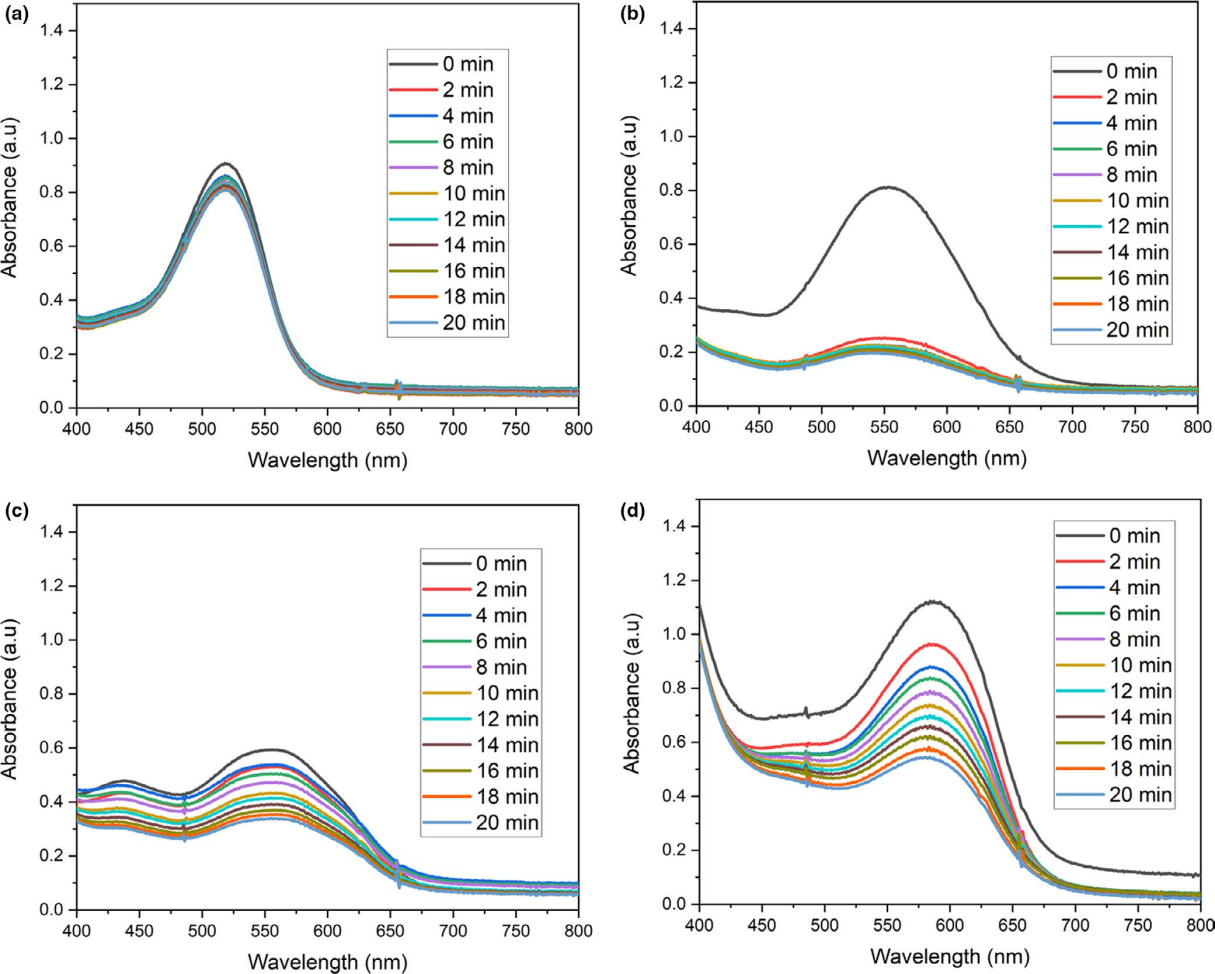
Photostability and bleaching rate of the dye extract (CM) of calyces of *Hibiscus sabdariffa* in different solutions and light illumination of 5 Mw/cm^2^. (a) Absorption spectra of the photostability study of the dye extract in dH2O. (b) Absorption spectra of the photostability study of the dye extract in Acetate‐HCl buffer (pH 4.6). (c) Absorption spectra of the photostability study of the dye extract in Phosphate Buffer Saline (pH 7.2). (d) Absorption spectra of the photostability study of the dye extract in Tris Base‐HCl buffer (pH 8.6). CM, cold methanol dye extract

The absorption spectra (Figure 2) indicating photostability and bleaching rate of the dye extract (CM) of the calyces of *H. sabdariffa* were determined in distilled water, Acetate‐HCL (AH) buffer (pH 4.6), Phosphate Buffer Saline (PBS; pH 7.2), and Tris Base‐HCL (TBH) buffer (pH 8.6). The dye extract is very photostable in water and do not bleach (Figure 2a). However, the dye‐extract bleaches in all the buffers used in this study (Figure 2b–d). The bleaching rate is faster in AH Buffer compared with other buffers under the same conditions of illumination with light (Figure 2b–d).

### 3.3 Mass spectroscopy of the dye extracts of the calyces of *H. sabdariffa*

The mass spectroscopy of the dye extracts was determined in order to assess the effects of extraction temperatures and solvents on the compositions of the extracts. The first three peaks (for positive and negative ionization modes) with the highest intensities for each of the samples are shown in Table 3 (see Figure S1A–G). The peaks indicated that the temperature and solvent of extraction did not affect the compositions of the dye extracts (Table 3). However, boiling at 100°C might have affected the compositions slightly (Table 3). The mass spectroscopy of the fractions of the dye extract (CM) showed it is a complex compound with more than 40 compounds (see Figure S2A and B).

**TABLE 3:** Major peaks of the mass spectroscopy of the dye extracts of calyces of *Hibiscus sabdariffa*

Dye extract	Peaks (*m*/*z*)					
	Positive			Negative		
	1st	2nd	3rd	1st	2nd	3rd
CM	118.080	203.051	245.024	189.0059	379.0166	407.0451
HM	118.079	245.025	203.051	189.0048	379.0153	407.0465
CMA	203.052	118.085	245.026	189.0062	379.0172	221.0316
HMA	118.084	203.052	245.026	189.0066	379.0172	351.0578
CW	118.086	597.146	219.027	189.0038	207.0145	351.0566
HW	203.053	118.084	219.027	189.0079	379.0169	207.0182
BW	203.052	383.1152	118.085	189.0051	210.9869	353.0872

Abbreviations: BW, boiling distilled water dye extract; CM, cold methanol dye extract; CMA, cold acidified methanol dye extract; CW, cold distilled water dye extract; HM, hot methanol dye extract; HMA, hot acidified methanol dye extract; HW, hot distilled water dye extract.

### 3.4 Photodynamic inactivation of *E. coli* and *B. subtilis* using the dye extracts of the calyces of *H. sabdariffa*

#### 3.4.1 Effect of buffer/solution on the PDI of bacteria using the dye extracts

**FIGURE 3:**
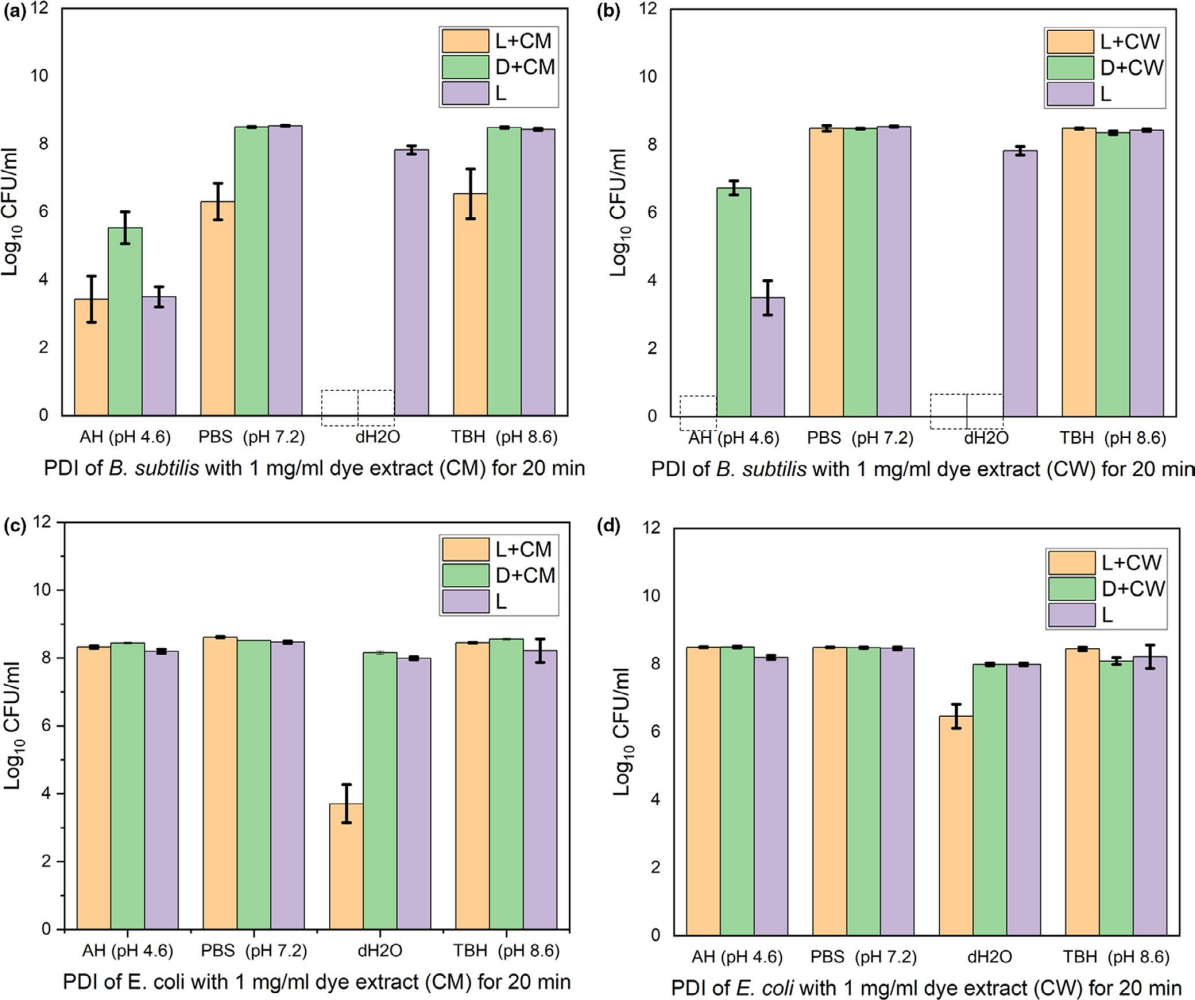
PDI of model bacteria using the dye extracts (CM and CW) with different buffers/solutions, illumination at 10 mW/cm^2^ for 20 min. (a) and (b) are PDI of *B. subtilis* using dye extracts CM and CW, respectively. (c) and (d) are PDI of *E. coli* using dye extracts CM and CW, respectively. AH, acetate‐HCl buffer; CM, cold methanol dye extract; CW, cold distilled water dye extract; dH_2_O, distilled water; PBS, phosphate buffer saline; TBH, Tris base‐HCl buffer; L+CM and L+CW are light experiments (dye extracts with light illumination); D+CM and D+CW are dark control experiments (dye extracts without light illumination); and L are “light only” control experiments (no dye extracts but illuminated with light). Data are mean ± *SD* (*n* = 3). Error bars show ±*SD*. 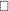, Complete inactivation

The PDI of *E.coli* and *B. subtilis* using the 1 mg/ml of dye extracts (CM and CW) was determined in distilled water (dH_2_O), Acetate‐HCl (AH) Buffer (pH 4.6), Phosphate Buffer Saline (PBS; pH 7.2), and Tris Base‐HCl (TBH) Buffer (pH 8.6). The light experiments (dye extract with light illumination) and “light only” control experiments were illuminated at 10 mW/cm^2^ for 20 min. The PDI of the model bacteria varies with different buffers/solutions used for the PDI (Figure 3). For both the dye extracts (CM and CW) used, the PDI was only clearly observed in dH_2_O and more *B. subtilis* was inactivated compared with the *E. coli* under the same conditions (Figure 3). In dH_2_O, both the PDI and non‐PDI were observed and could not be differentiated in *B. subtilis* as both the light experiments (dye extract with light illumination) and the dark experiments (dye extracts without illumination) lead to the complete inactivation (limit of detection) equivalent to reductions of 8.5 log CFU/ml (Figure 3a,b). Also, inactivation (reductions of 1.5–8.5 log CFU/ml) including the controls were observed for *B. subtilis* in AH Buffer (pH 4.6; Figure 3a,b). As for the PDI in the remaining buffers, inactivation – most likely PDI was observed only when the dye extract (CM) was used for *B. subtilis* in PBS (pH 7.2) and TBH Buffer (pH 8.6) with the reductions of 1.7 log CFU/ml and 1.5 log CFU/ml, respectively (Figure 3a,b). As for the *E. coli*, PDI was only observed in dH_2_O with reductions of 4.8 log CFU/ml and 2.2 log CFU/ml for dye extracts CM and CW, respectively (Figure 3c,d).

#### 3.4.2 Effect of the concentration and time of illumination on PDI using the dye extracts

**FIGURE 4:**
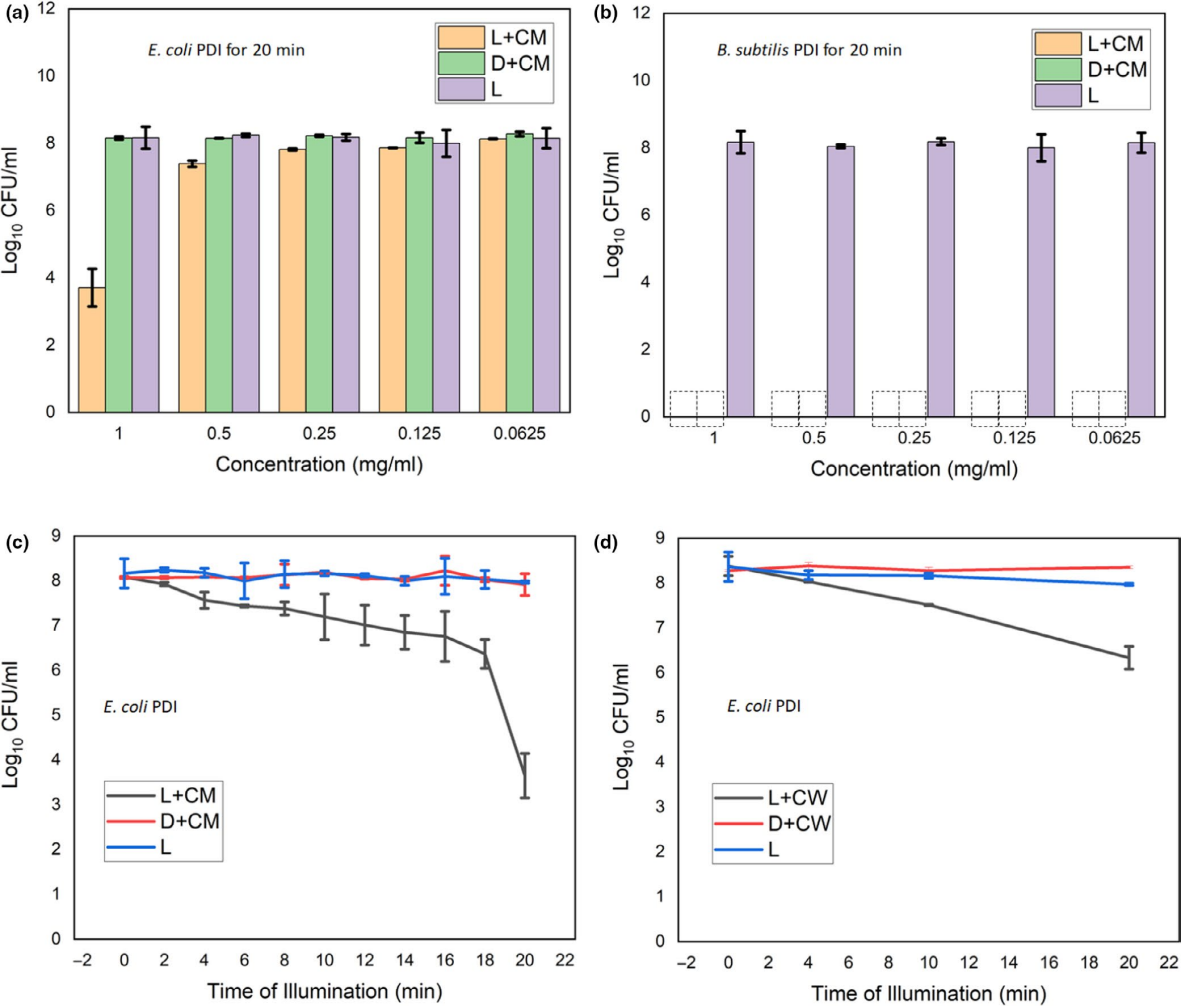
PDI of model bacteria using the dye extracts (CM and CW) at different concentrations and different time of illumination at 10 Mw/cm^2^. (a) PDI of *E. coli* using dye extracts CM at different concentrations 0.0625–1 mg/ml. (b) PDI of *B. subtilis* using dye extracts CM at different concentrations 0.0625–1 mg/ml. (c) PDI of *E. coli* using dye extracts CM at different illumination times from 2 to 20 min. (d) PDI of *E. coli* using dye extracts CW at different illumination times from 2 to 20 min. CM, cold methanol dye extract; CW, cold distilled water dye extract; L + CM and L + CW are light experiments (dye extracts with light illumination); D + CM and D + CW are dark control experiments (dye extracts without light illumination); and L are “light only” control experiments (no dye extracts but illuminated with light). Data are mean ± *SD* (*n* = 3). Error bars show ± *SD*. 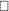, Complete inactivation

**FIGURE 5:**
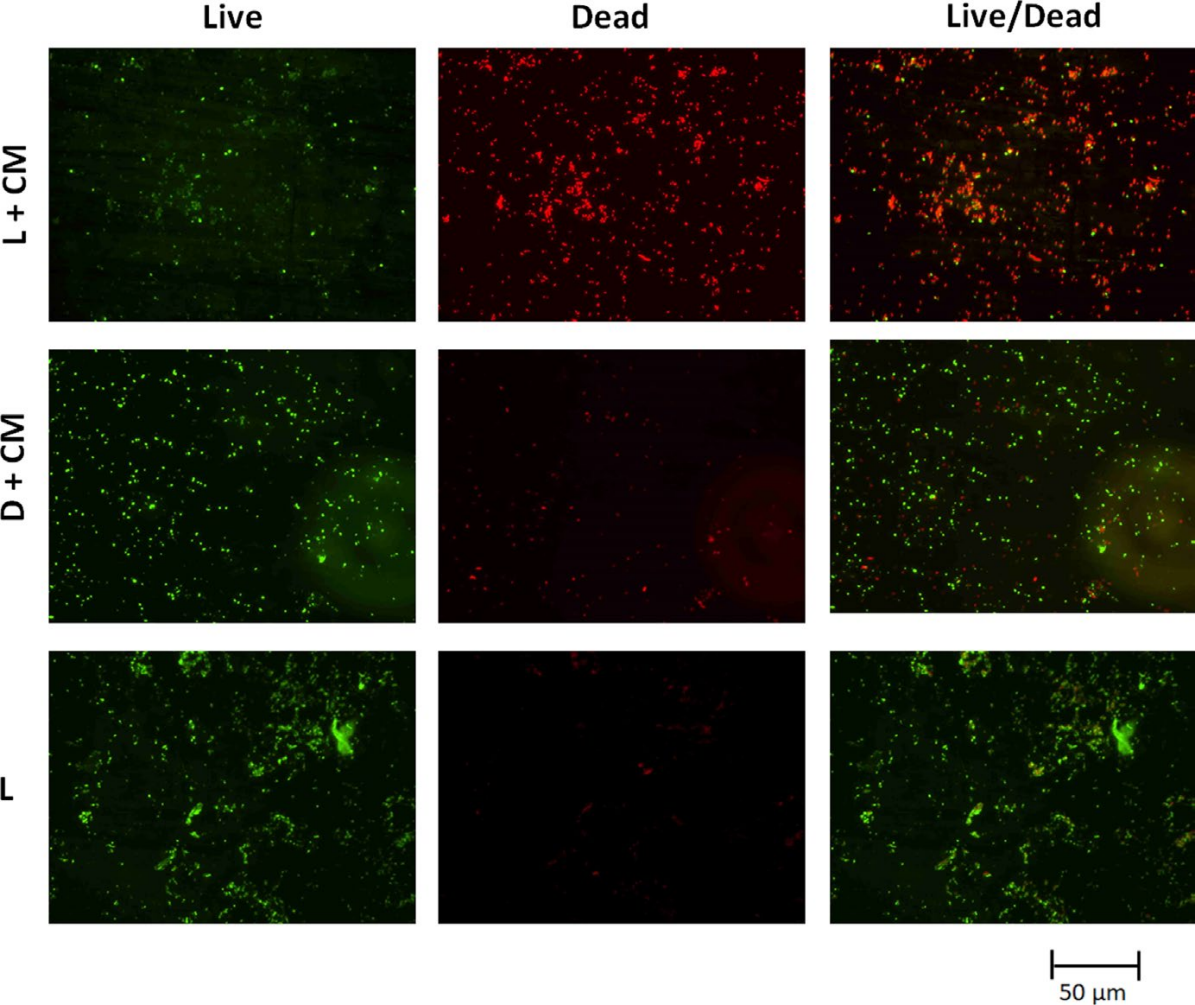
Fluorescence microscopy images of live/dead staining of the PDI samples of *E. coli* when 1 mg/ml of the dye extract–CM was used in water. CM, cold methanol dye extract. L + CM is the light experiment (dye extract with light illumination at 10 mW/cm^2^ for 20 min); D + CM is the dark control experiments (dye extract without light illumination for 20 min); and L is “light only” control experiment (no dye extracts but illuminated with light at 10 mW/cm^2^ for 20 min)

**FIGURE 6:**
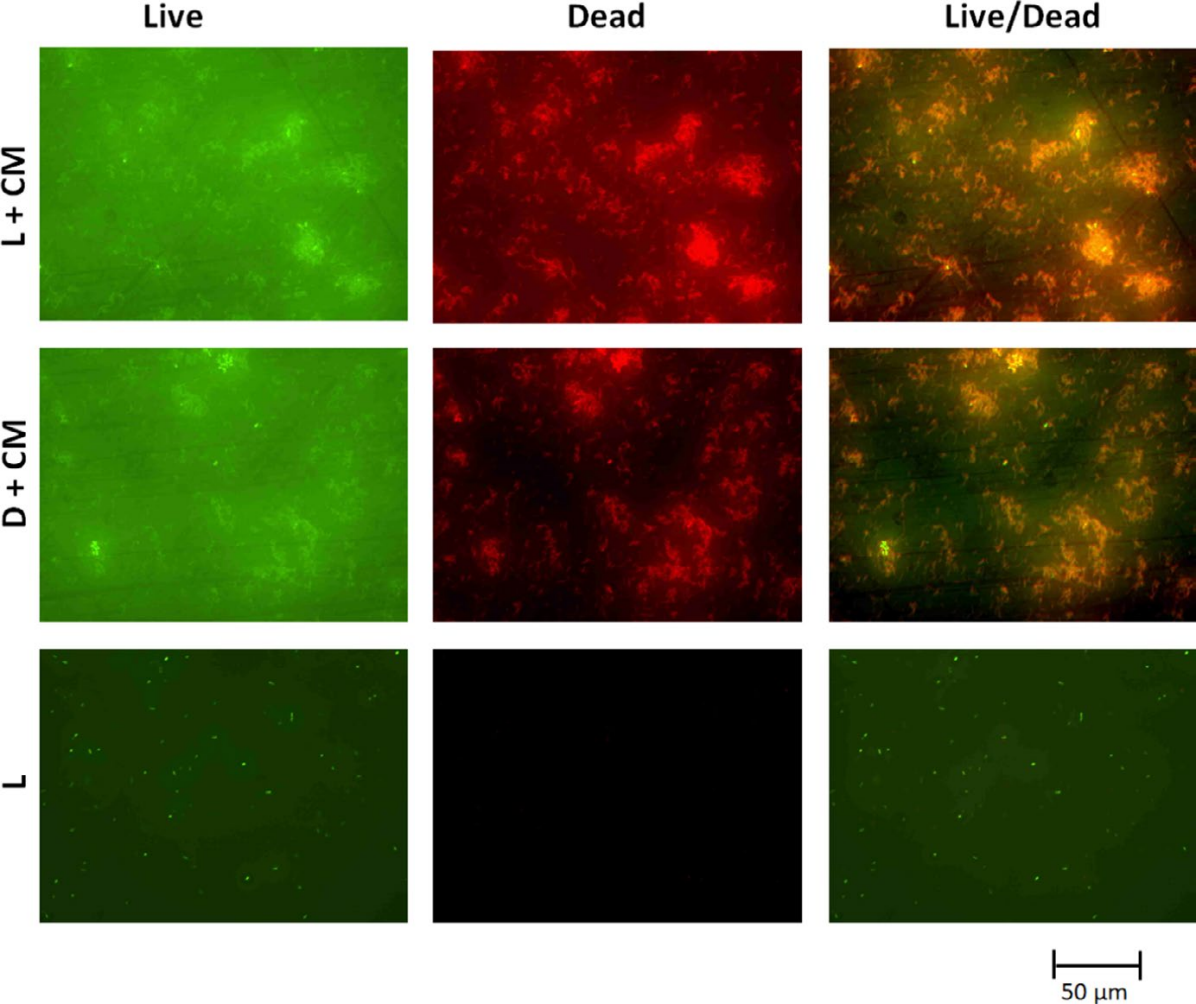
Fluorescence microscopy images of live/dead staining of the PDI samples of *B. subtilis* when 1 mg/ml of the dye extract (CM) was used in water. CM, cold methanol dye extract. L + CM is the light experiment (dye extract with light illumination at 10 mW/cm^2^ for 20 min); D + CM is the dark control experiments (dye extract without light illumination for 20 min); and L is “light only” control experiment (no dye extracts but illuminated with light at 10 mW/cm^2^ for 20 min)

The PDI of *E.coli* and *B. subtilis* using different concentrations of the dye extracts (CM and CW) and different time of illumination at 10 mWcm^‐2^ were determined in dH_2_O. We observed dose/concentration and illumination time‐depended inactivation (Figure 4). For the 20 min PDI of *E. coli*, reductions of 4.7, 1.2, 0.7, 0.6, and 0.4 log CFU/ml were observed for the 1, 0.5, 0.25, 0.125, and 0.0625 mg/ml of the dye extract (CM), respectively (Figure 4a). As for the *B. subtilis*, complete inactivation (reductions of 8.5 log CFU/ml) was observed for both the light experiments (dye extract with light illumination) and the dark experiments (dye extract without light illumination) for the all the concentrations (0.0625–1 mg/ml) of the dye extract (CM) used (Figure 4b). The illumination time‐depended study of the *B. subtilis* also showed complete inactivation (reductions of 8.5 log CFU/ml) for both the light experiments (dye extract with light illumination) and the dark experiments (dye extract without light illumination) for all the time 2–20 min tested (Results not shown). However, we were able to follow and observed the illumination time (2–20 min)‐depended PDI of *E. coli* for the dye extracts CM and CW (Figure 3c,d). For the PDI using the dye extract (CM), at times 4, 10, and 20 min of PDI, we observed reductions of 1, 1.3, and 4.8 log CFU/ml, respectively, of the *E. coli* (Figure 4c). However, using the dye extract (CW), at time 4, 10 and 20 min of PDI, we observed reductions of 0.5, 1, and 2.2 log CFU/ml, respectively, of the *E. coli* (Figure 4d). The fluorescence microscopy images of the live/dead staining of the PDI samples validated the extent of inactivation using the dye extract (CM) in water (Figures 5 and 6).

## 4 DISCUSSION

### 4.1 Extraction and photophysical characteristics of the dye extracts of calyces of *H. sabdariffa*: implications for applications

In this work, we extracted the dye from the calyces of *H. sabdariffa* using different solutions; distilled water, methanol and acidified methanol, and different temperatures; cold, hot, and boiling temperatures. Most earlier reports suggested the use of acidified methanol/ethanol at cold and/or hot temperatures for the extractions of the dyes (Abou‐Arab et al., 2011; Ilori & Odukoya, 2005). However, we also used water for the extraction of the dye of calyces of *H. sabdariffa* at cold, hot, and boiling temperatures because of the almost universal availability of water even in the remotest part of the world. For all the solvents and temperatures used, the % yield of the dried dye extracts ranges from 25 to 40 (Table 1). The water dye extractions had higher yield than the methanol and acidified methanol extractions (Table 1). However, at the same concentration (1 mg/ml) the water dye extracts were a little bit less acidic (pH 3.0) when compared with the methanol (pH 2.85) and acidified methanol (pH 2.76) extracts (Table 1). These pH (2.76–3.0) can inactivate many of the bacteria especially the Gram‐positive bacteria. The pH of the acidified methanol dye extract we observed agrees with the earlier reported (Abou‐Arab et al., 2011). Using the same solvent but different temperatures for the dye extracts does not significantly affect the % yields and the pH. All these indicated the ease at which the dyes of calyces can be extracted even in cold water with no additional need for energy for heating thereby making it suitable for the aim of this project.

The absorption and mass spectra (except for the dye extract–BW) indicated that irrespective of the solvents and temperatures of extractions, the dye extracts are the same (Figure 1a and Table 3). The absorption peak for the dye extracts is the same which is 520 nm. This wavelength is well within the visible range and it means sunlight (with spectrum of 300–1,400 nm and a peak at 500–600 nm; Jemli, Alouini, Sabbahi, & Gueddari, 2002; Loeb, Hofmann, & Kim, 2016) could be used to irradiate the dye for the photodynamic inactivation of fresh produce spoilage and pathogenic microorganisms. However, the absorption peaks and colors of the dye extracts were pH/buffer dependent (Figure 1b,c)—this is well‐known characteristics of the anthocyanins especially the types that are found in the calyces of *H. sabdariffa* (Abou‐Arab et al., 2011; Ilori & Odukoya, 2005).

The dye extract of the calyces of *H. sabdariffa* is very photostable and pH stable in water (Figure 2a and Table 2). This means that the dye extract has fulfilled one of the cardinal desired characteristics of a photosensitizer for the lack of photodegradation and bleaching in water (DeRosa & Crutchley, 2002). However, the dye extracts bleaches in other buffers (acetate‐HCl buffer pH 4.6, PBS pH 7.2, and tris base‐HCl pH 8.6) used (Figure 2b–d). The bleaching may be pH and/or buffer type/compositions dependent. This may limit the application of the dye extracts as a photosensitizer to only environmental applications such as fresh produce sanitation where fresh water would be used as wash waters containing the dye extracts to photodynamically inactivate pathogenic and spoilage microorganisms. The dye extract may not be suitable for the PDI in biological samples and solutions/buffers of high salts contents. However, the non‐PDI inactivation activity of the dye extracts could still be available or retained and could be exploited for the biological samples and solutions/buffers.

### 4.2 Dye extracts of the calyces of *H. sabdariffa* cause photodynamic and nonphotodynamic inactivation of *E. coli* and *B. subtilis*

The water and methanol dye extracts of the calyces of *H. sabdariffa* used in this work showed both photodynamic and nonphotodynamic inactivation of bacteria models (Figures 3 and 4). The photodynamic inactivation observed depends on the buffers/solutions of the PDI and was clearly observed in dH_2_O. This follows and may be linked to the photostability of the dye extracts in dH_2_O (Figure 2a) which allowed the molecules of dye at ground state to absorb the illuminated light and thereby become excited to singlet state then to triplet state which can then interact with the available molecular oxygen to generate singlet‐oxygen and other reactive oxygen species which are cytotoxic and can inactivate microorganisms including bacteria in solution. Singlet‐oxygen and other ROS oxidize and cause irreversible damage to proteins, lipids, nucleic acid, and other cellular components of microorganisms and ultimately inactivate them (Costa et al., 2013).

For the photosensitizers that are photostable including the dye extracts of the calyces of *H. sabdariffa* in water showed in this work (Figure 2a), the triplet state of their excited molecules interacts with the available molecular oxygen which ultimately lead to the production of the ROS. After the interaction with the oxygen, the molecules of the photosensitizers will go to the ground state to be excited again with the illuminated light and the process continues with the resultant production of ROS. However, for the photosensitizers that are not photostable including the dye extracts of calyces of *H. sabdariffa* in acetate‐HCl buffer (pH 4.6), phosphate buffer saline (pH 7.2), and tris base‐HCl buffer (pH 8.6) observed in this work (Figure 2b–d), at each interactions of the excited triplet state of the molecules of the photosensitizers with the molecular oxygen, the ROS produced especially the singlet oxygen, consumed the molecules of the photosensitizers that produces them thereby leading to the bleaching and photodegradation of the photosensitizers/dyes. This led to the steady reduction in the molecules of the photosensitizers which also corresponds to steady reduction in the ROS generation in the solution. The generation of the ROS in the solution would eventually stop within seconds to few minutes. The insufficient ROS production in solution, ROS produced degrading the molecules of photosensitizers that produces them instead of oxidizing the bacteria in the solution, and the eventual stoppage of the production of ROS maybe the plausible reasons for the lack or less bacteria PDI observed in this study when acetate‐HCl buffer (pH 4.6), phosphate buffer saline (pH 7.2), and tris base‐HCl buffer (pH 8.6) were used (Figure 3). We hypothesize that the polyphenols especially the anthocyanins in the dye extracts of the calyces of *H. sabdariffa* may be responsible for the PDI of bacteria observed in this work. To the best of our knowledge, the photodynamic antibacterial activity as shown in this work has never been reported before for the dye extracts of the calyces of *H. sabdariffa*.

Non‐PDI was observed for *B. subtilis* in this study (Figures 3 and 4). This maybe attributable to the polyphenols especially the organic acids such as citric acid, hydroxycitric acid, hibiscus acid, malic acid, tartaric acid, oxalic acid, and ascorbic acid in the dye extracts (Da‐Costa‐Rocha et al., 2014). The dye extracts cause the water to be acidic (pH 2.85–3.85) and this, in addition to the antibacterial activity of other compounds present in the extracts, maybe responsible for the complete inactivation of *B. subtilis* for both the light experiments and dark control experiments in water observed in this work (Figures 3 and 4). *B. subtilis* used in this study was particularly sensitive to acetate‐HCl buffer (pH 4.6) and the buffer with its low pH might have induced some molecules within the bacterium to act as photosensitizers even without the addition of the external photosensitizers to generate ROS which inactivated it (Figure 3a,b).

Like most antimicrobial agents, the PDI and non‐PDI activity of the dye extracts of calyces of *H. sabdariffa* shown in this work are dye dose/concentration and illumination time dependent (Figure 4). Previous reports have shown that the PDI is proportionally related to the light dose/fluence rate (Costa et al., 2010; Majiya, 2017). The light intensity used for the PDI in this work is 10 mW/cm^2^ which is just about 1% of the bright sunlight under clear sky conditions in sub‐Saharan Africa. In this work, under the same conditions, more *B. subtilis*–a Gram‐positive bacteria was inactivated compared with the *E. coli*–a Gram negative. This is a general trend for most of the antibacterial agents including the PDI. Gram‐positive bacteria are easily inactivated by singlet‐oxygen oxidation as compared with gram‐negative bacteria due to differences in the structure of their cell membrane (Alves et al., 2009; Bourré et al., 2010; Carvalho et al., 2007; Costa et al., 2012; Komagoe et al., 2011; Maisch, Spannberger, Regensburger, Felgenträger, & Bäumler, 2012). Gram‐negative bacteria have an additional outer membrane apart from the cytoplasmic (inner) membrane, giving them extra protection against antimicrobial agents including singlet‐oxygen and other ROS produced during photosensitization.

## 5 CONCLUSION

In summary, we observed that the photostability, photodynamic, and nonphotodynamic antibacterial activity of the water and methanol dye extracts of the calyces of *H. sabdariffa* were dependent on the solutions/buffers, dye dose/concentration, and illumination time. The dye extracts were very photostable in distilled water. However, the dye extracts bleached and photodegraded in acetate‐HCl buffer (pH 4.6), PBS (pH 7.2), and tris base‐HCl buffer (pH 8.6). This will limit the PDI use of the dyes to only environmental applications such as fresh produce sanitation in fresh water. The PDI of bacteria was clearly observed in distilled water because the dye extracts were photostable in the water. To the best of our knowledge, this study will be the first time that photodynamic antibacterial activity of the dye extracts of calyces of *H. sabdariffa* is reported. Rate and extent of the PDI and non‐PDI was higher in *B. subtilis* compared with the *E. coli*. This study can lead way to the development of a low‐tech sunlight‐driven fresh produce sanitation system that could be used in developing countries especially the sub‐Saharan African to reduce foodborne diseases/outbreaks and fresh produce spoilage/wastage.

## CONFLICT OF INTEREST

None.

## ETHICAL CONSIDERATION

Ethics approval was not required for this research.

## Supporting information

Figure S1

Figure S2
