# Supplementary material for: Dye extract of calyces of Hibiscus sabdariffa has photodynamic antibacterial activity: A prospect for sunlight‐driven fresh produce sanitation
Source: Food Sci Nutr. 2020 Apr 21;8(7):3200–11. doi: 10.1002/fsn3.1580 (PMC7382145; doi:10.1002/fsn3.1580)
Supplement: Supplementary file 1 — Figure S1 [file FSN3-8-3200-s001.docx]

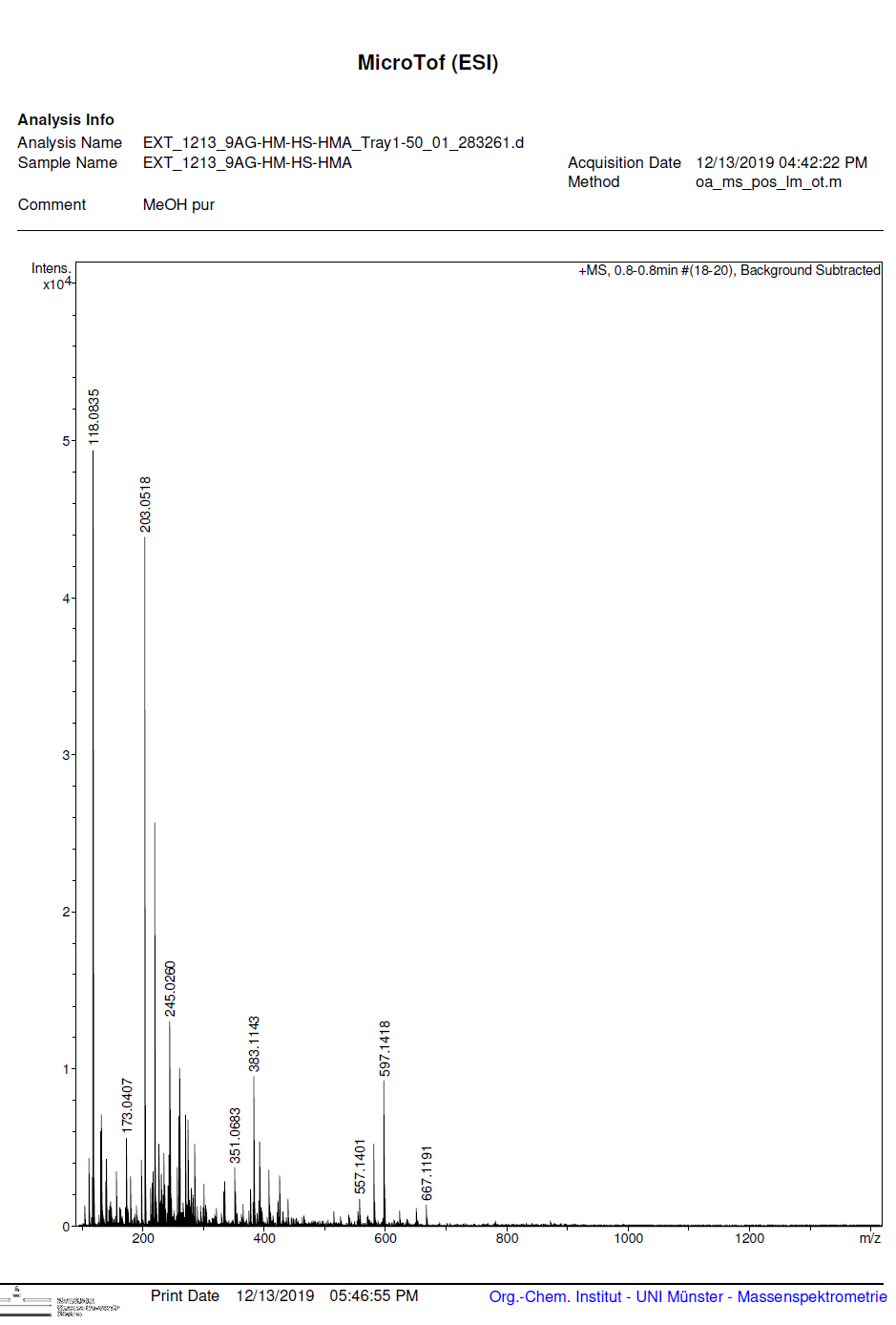


Figure S1 A: Mass spectroscopy of Hot Acidified Methanol (HMA) dye extract (positive ionisation mode)


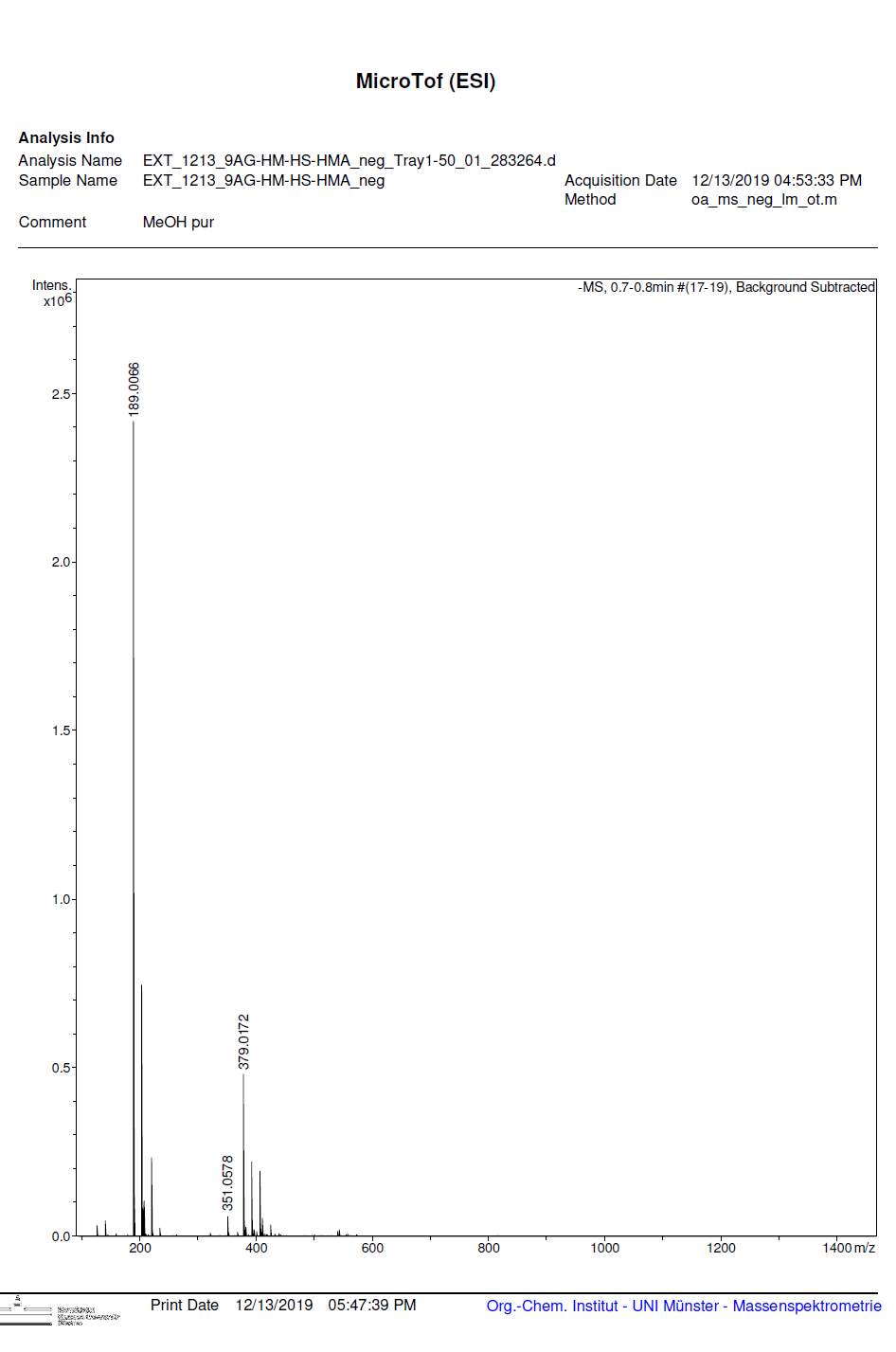


Figure S1 A: Mass spectroscopy of Hot Acidified Methanol (HMA) dye extract (negative ionisation mode)


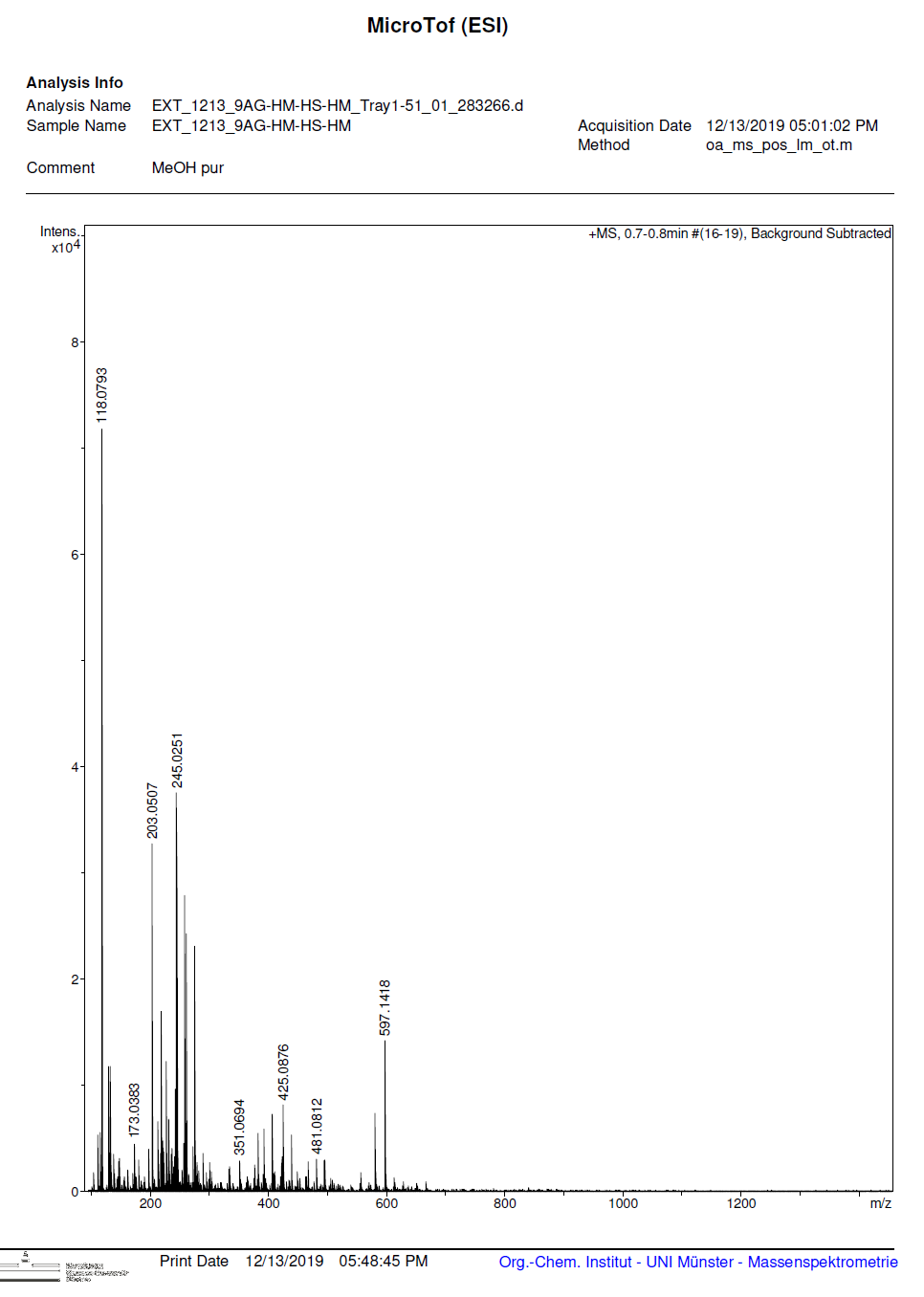


Figure S1 B: Mass spectroscopy of Hot Methanol (HM) dye extract (positive ionisation mode)


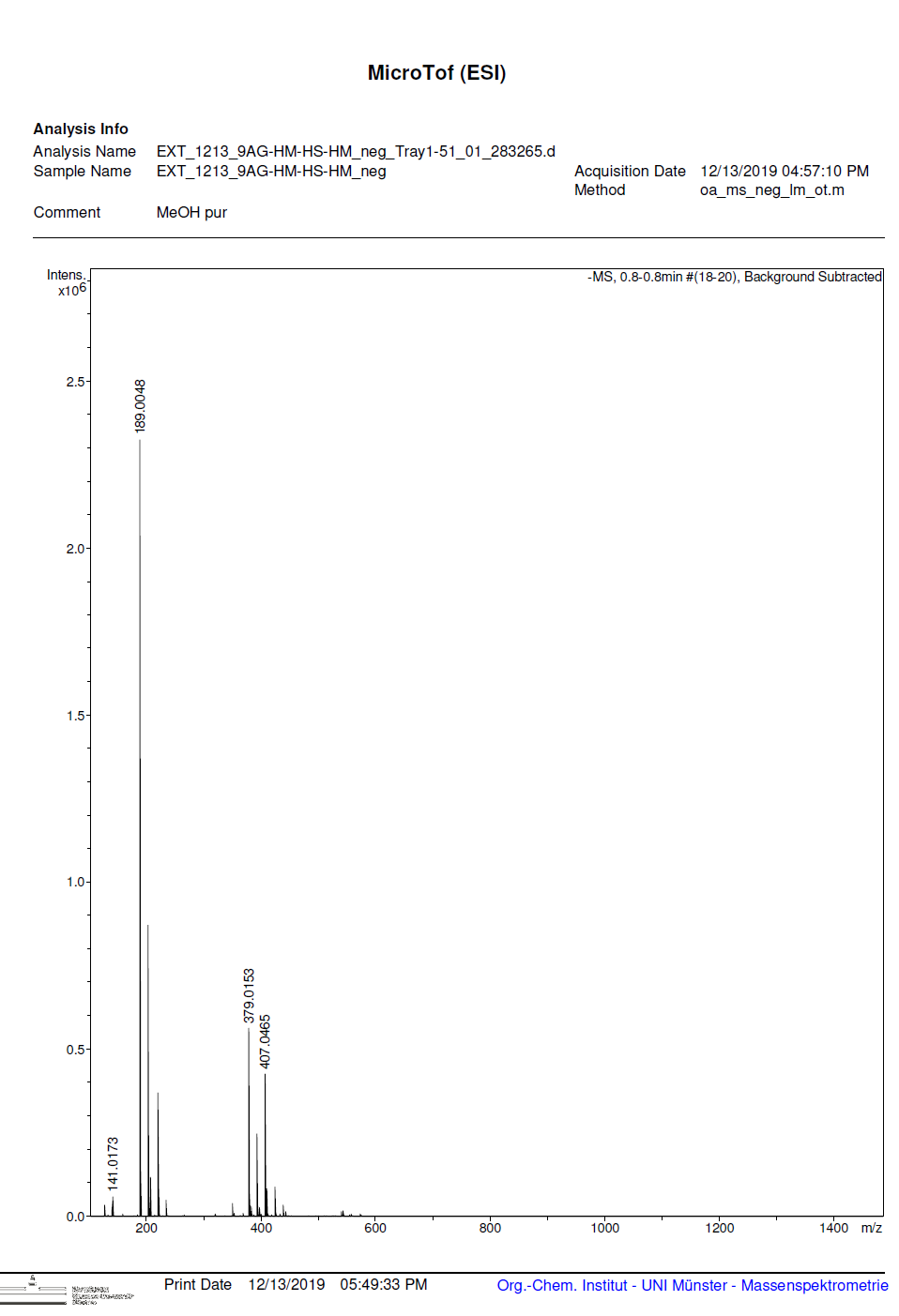


Figure S1 B: Mass spectroscopy of Hot Methanol (HM) dye extract (negative ionisation mode)


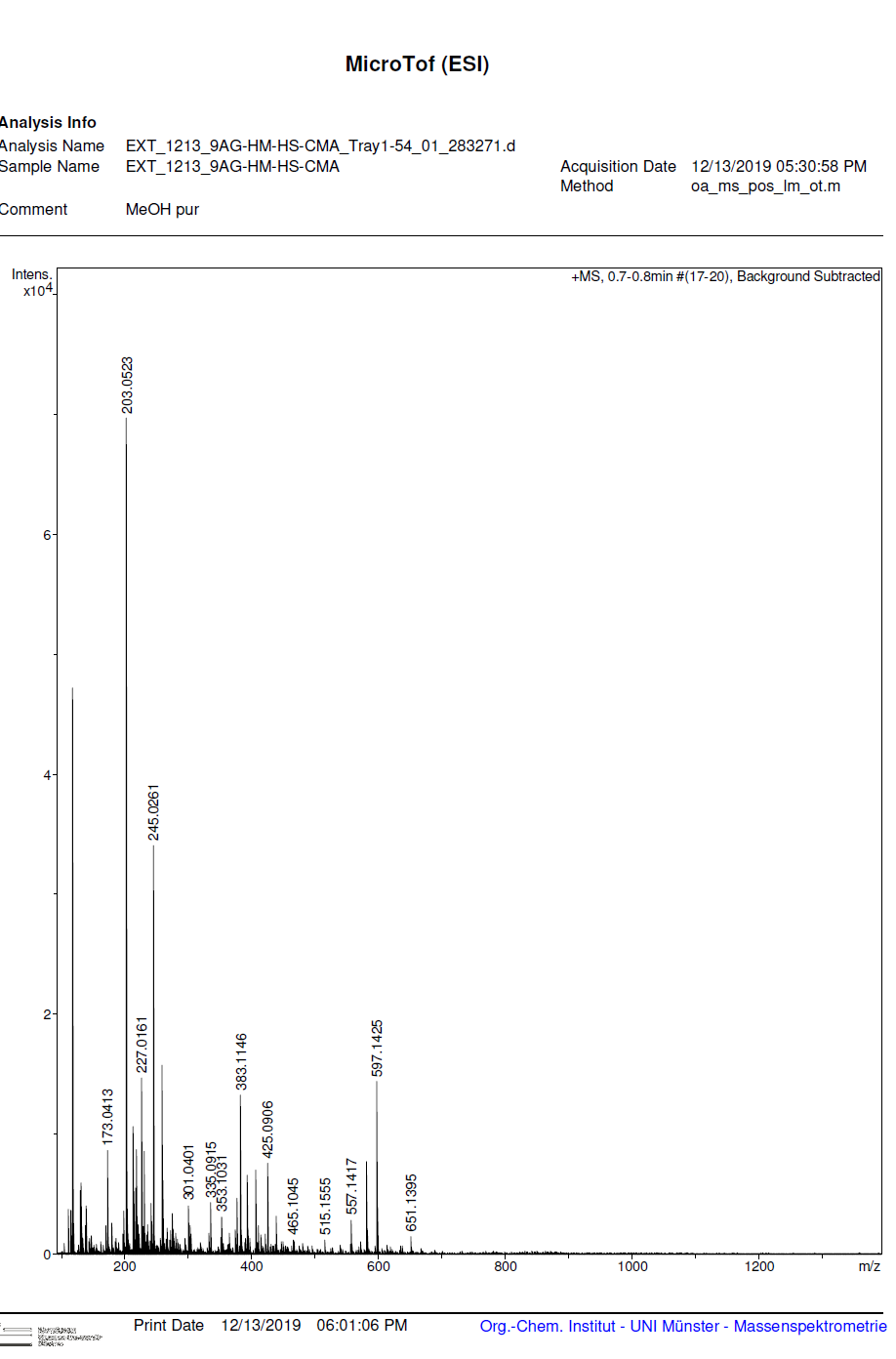


Figure S1 C: Mass spectroscopy of Cold Acidified Methanol (CMA) dye extract (positive ionisation mode)


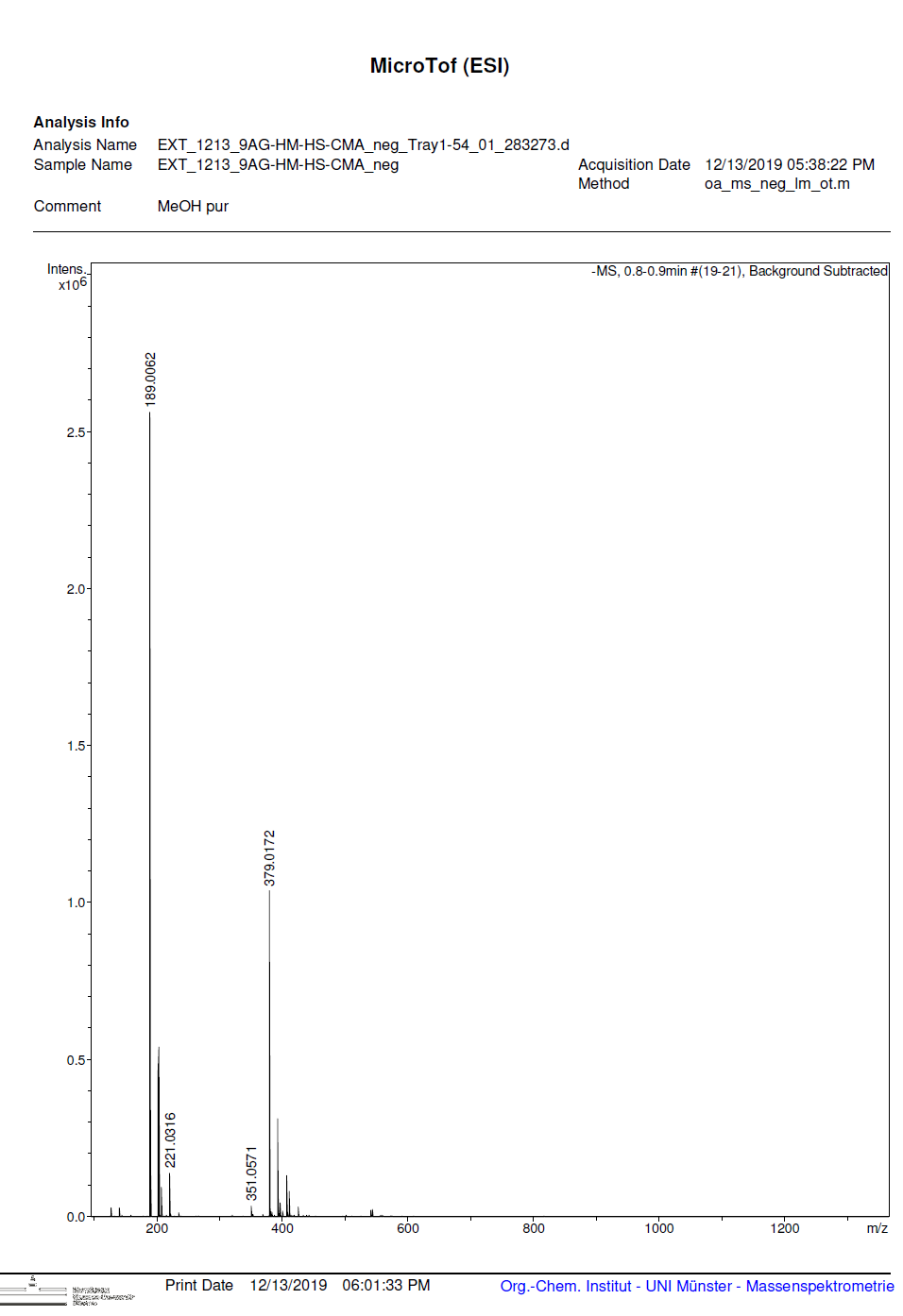


Figure S1 C: Mass spectroscopy of Cold Acidified Methanol (CMA) dye extract (positive ionisation mode)


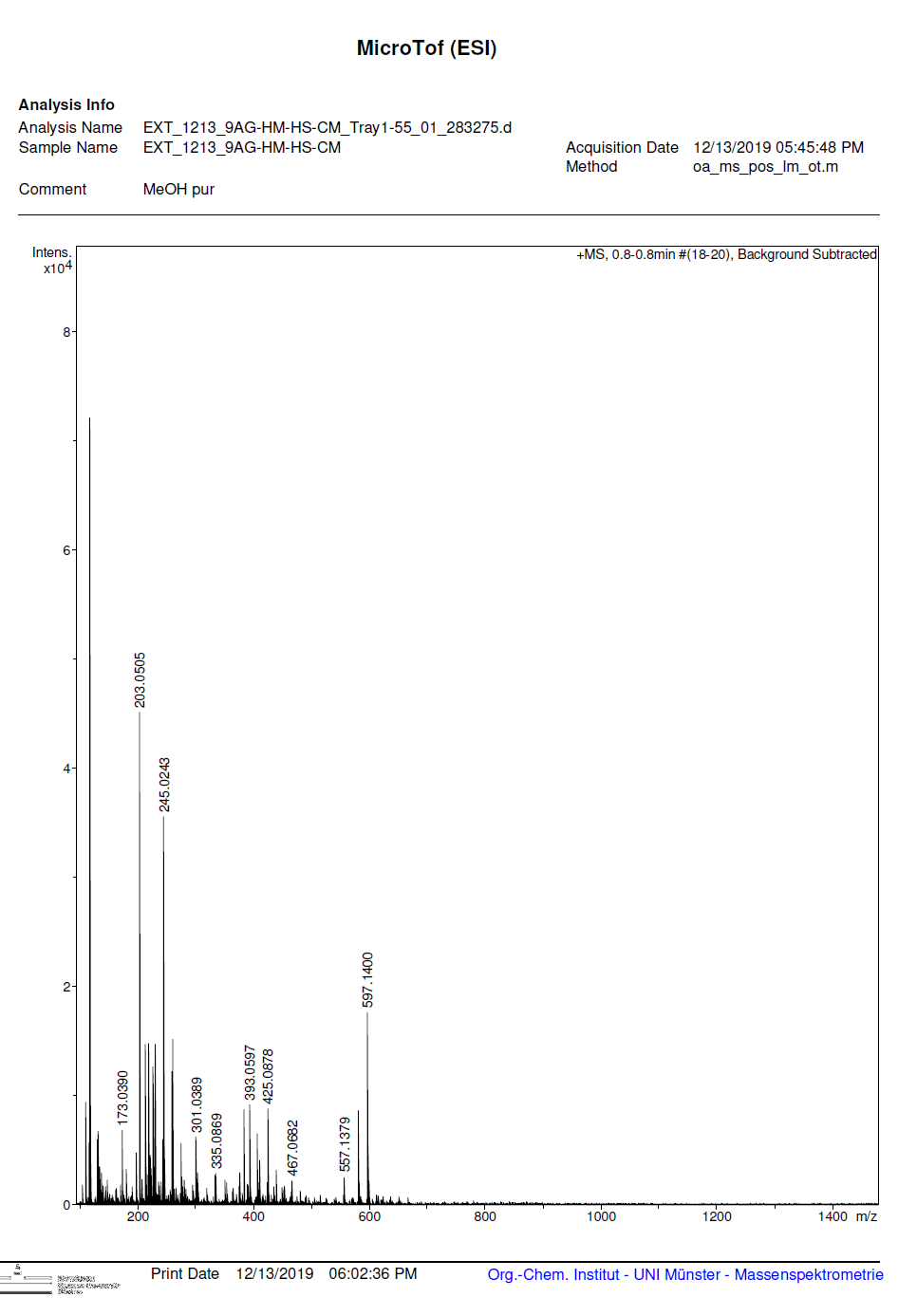


Figure S1 D: Mass spectroscopy of Cold Methanol (CM) dye extract (positive ionisation mode)


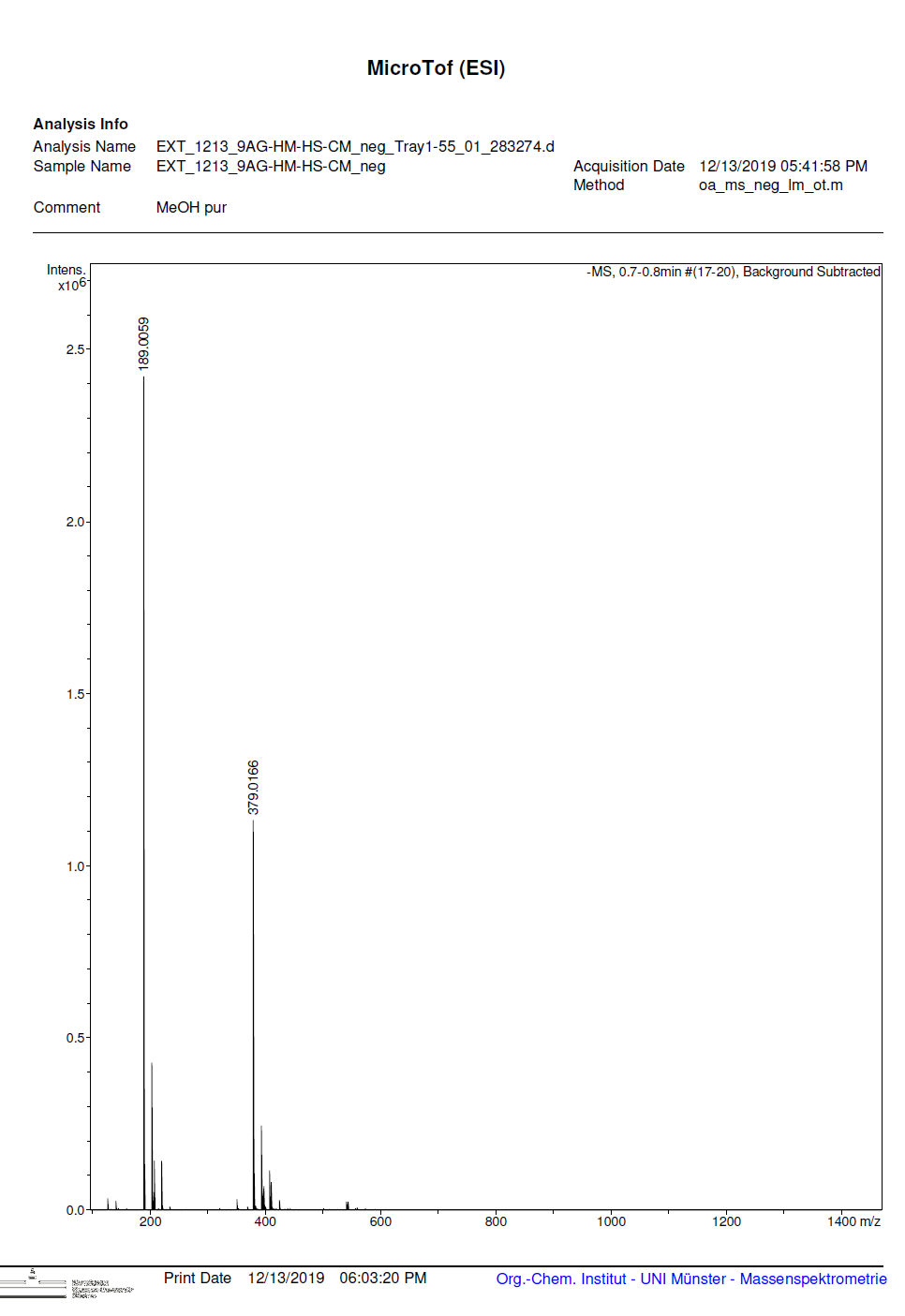


Figure S1 D: Mass spectroscopy of Cold Methanol (CM) dye extract (negative ionisation mode)


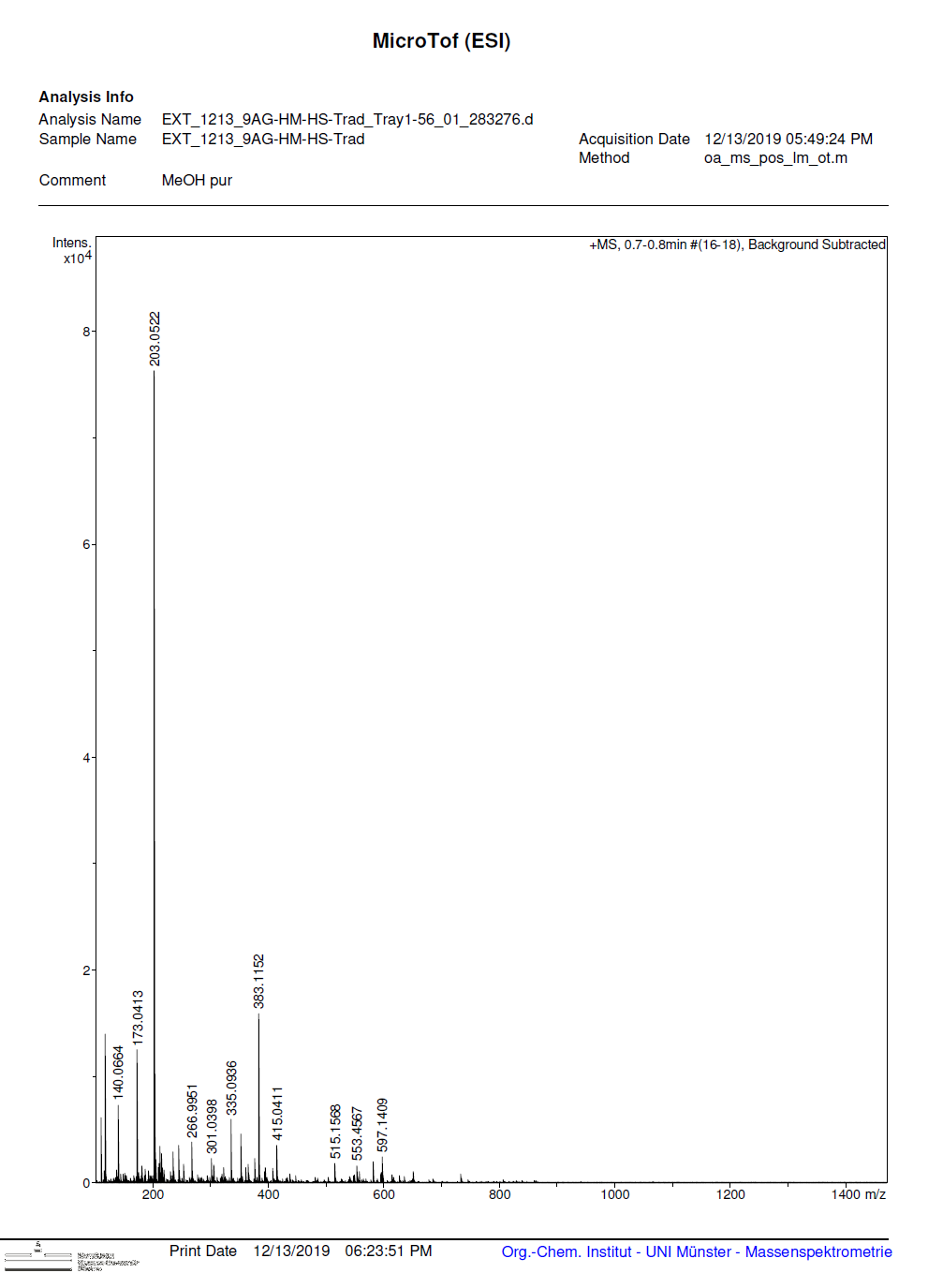


Figure S1 E: Mass spectroscopy of Boiling Water (BW) dye extract (positive ionisation mode)


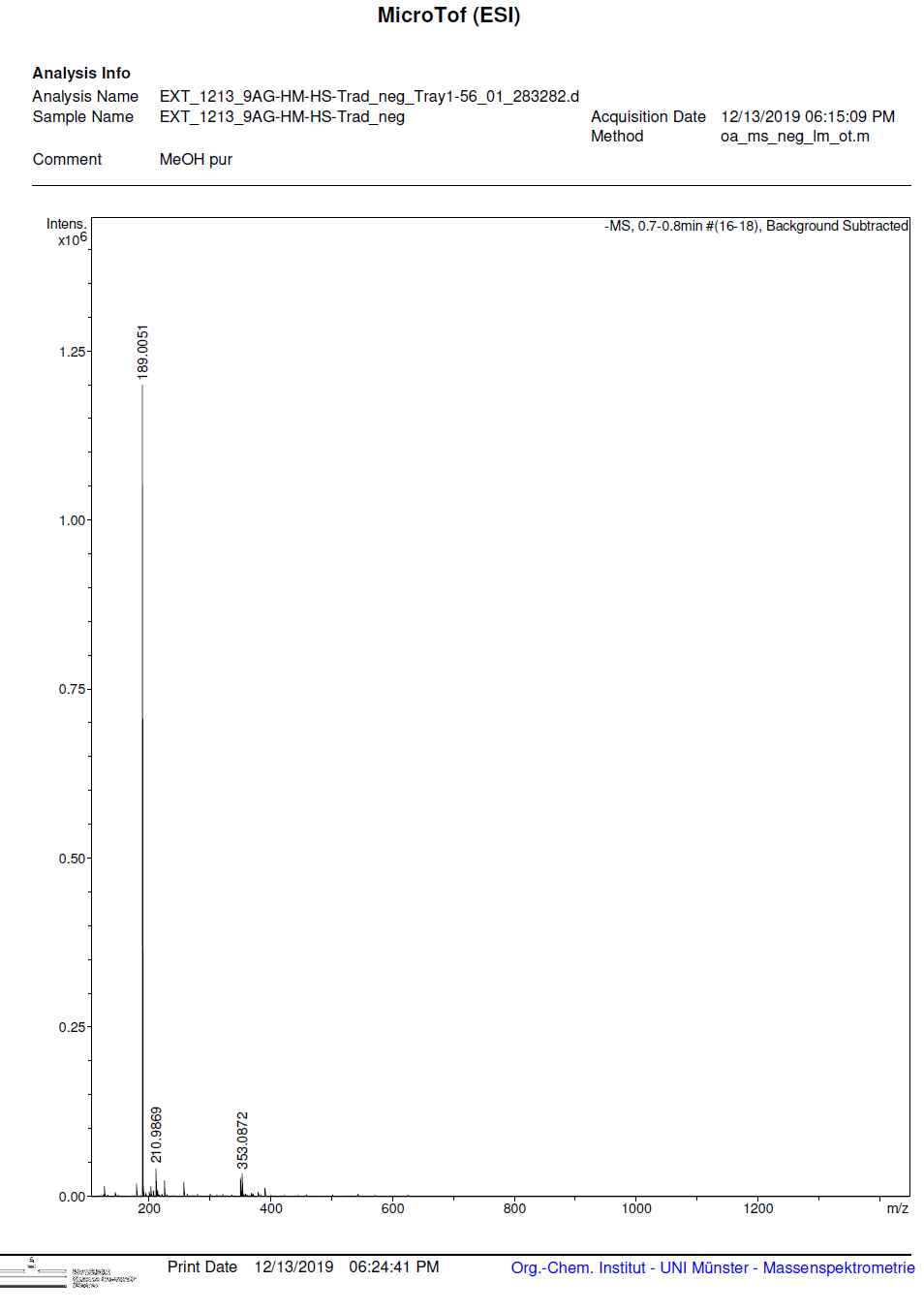


Figure S1 E: Mass spectroscopy of Boiling Water (BW) dye extract (negative ionisation mode)


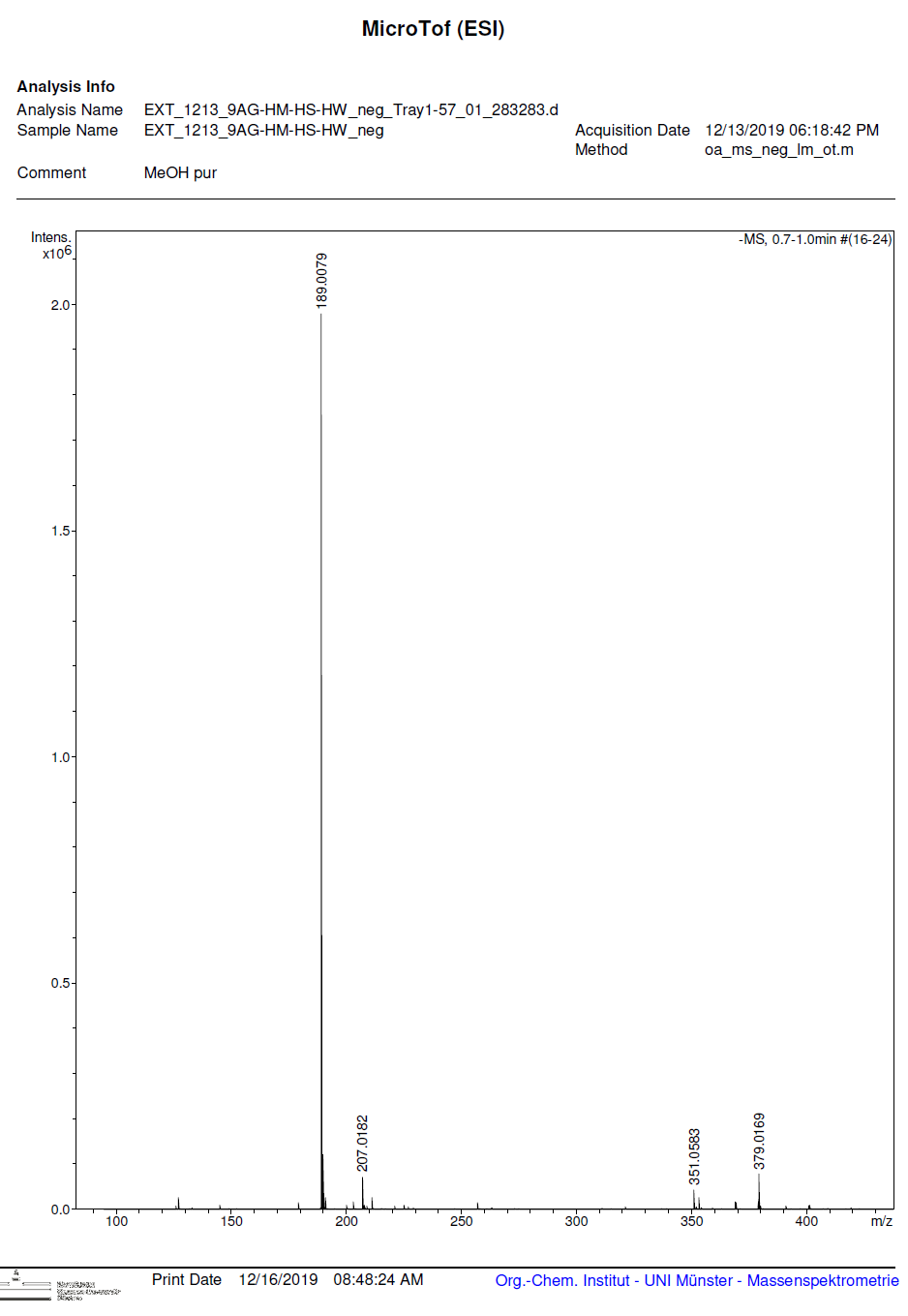


Figure S1 F: Mass spectroscopy of Hot Water (HW) dye extract (negative ionisation mode)


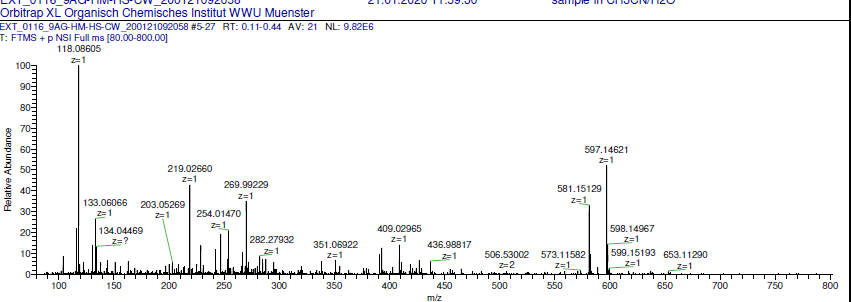


Figure S1 G: Mass spectroscopy of Cold Water (CW) dye extract (positive ionisation mode)


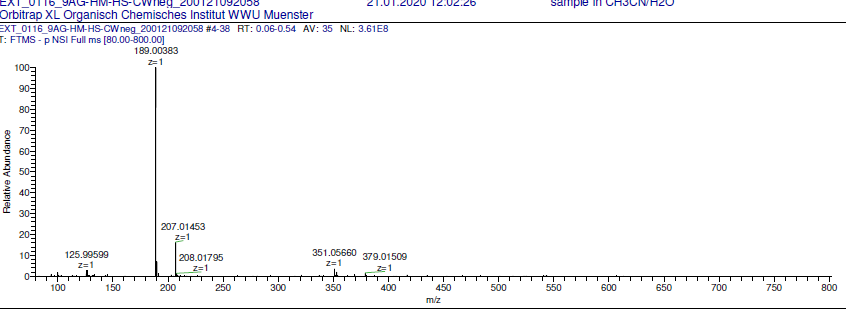


Figure S1 G: Mass spectroscopy of Cold Water (CW) dye extract (negative ionisation mode)
