# Supplementary material for: Dye extract of calyces of Hibiscus sabdariffa has photodynamic antibacterial activity: A prospect for sunlight‐driven fresh produce sanitation
Source: Food Sci Nutr. 2020 Apr 21;8(7):3200–11. doi: 10.1002/fsn3.1580 (PMC7382145; doi:10.1002/fsn3.1580)

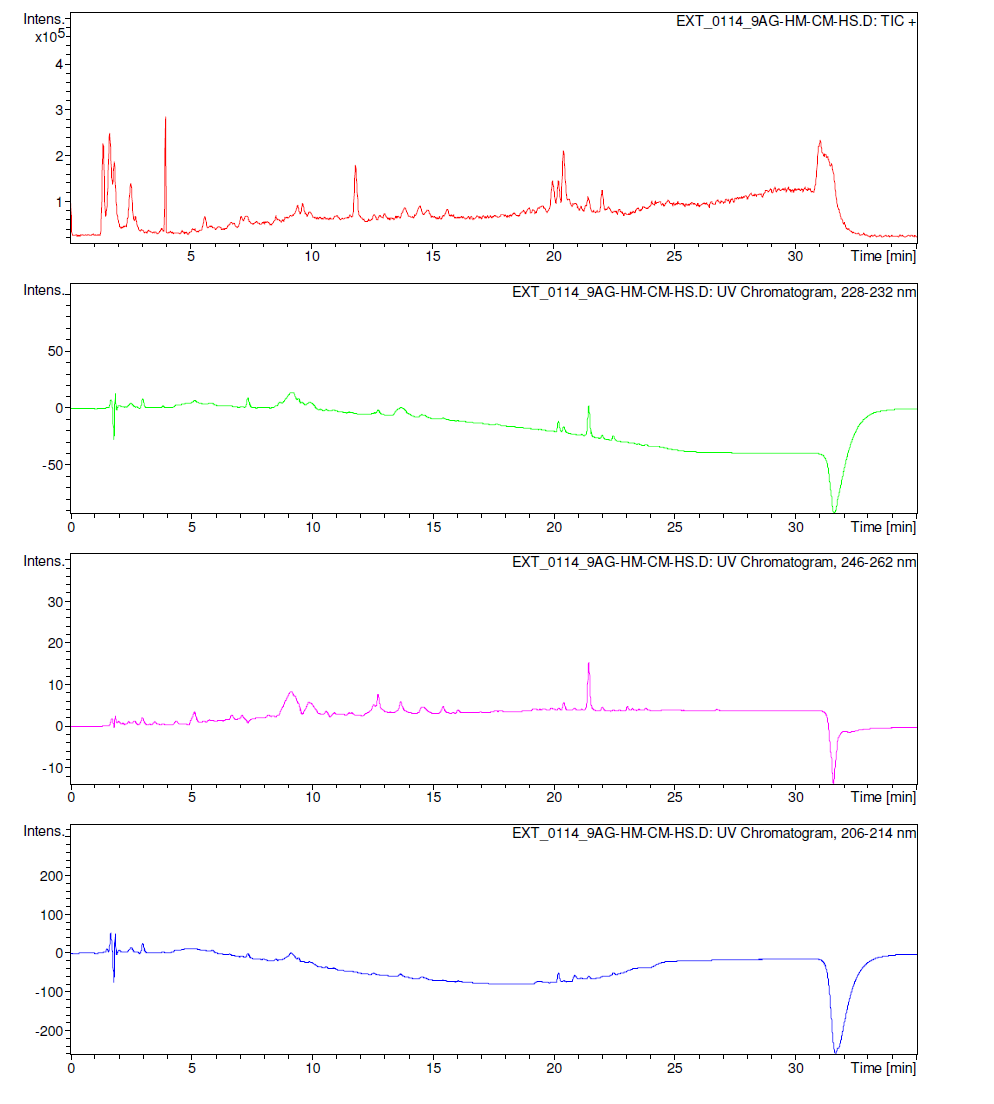


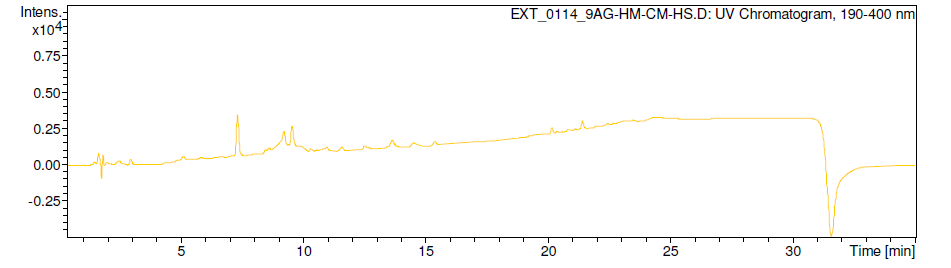


Figure S2 A: Chromatograms of Cold Methanol (CM) dye extract

Figure S2 B: Tables showing the Chromatograms dissects of Cold Methanol (CM) dye extract


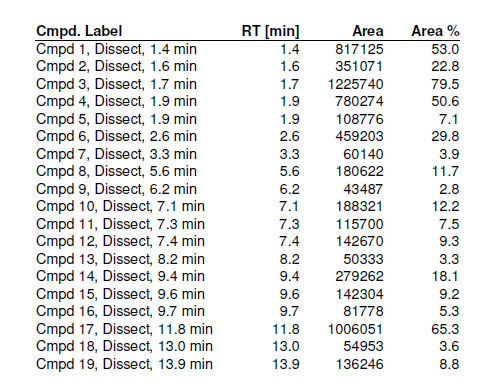


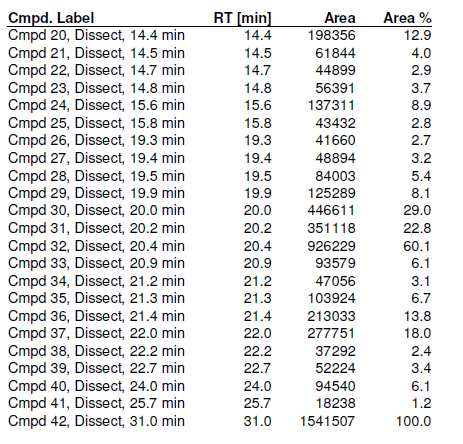

Supplement: Supplementary file 2 — Figure S2 [file FSN3-8-3200-s002.docx]
